# Supplementary material for: M2 macrophage-derived exosomal microRNA-155-5p promotes the immune escape of colon cancer by downregulating ZC3H12B
Source: Mol Ther Oncolytics. 2021 Feb 6;20:484–98. doi: 10.1016/j.omto.2021.02.005 (PMC7932913; doi:10.1016/j.omto.2021.02.005)
Supplement: Document 1. Figures S1–S7 and Table S1 [file mmc1.pdf]

OMTO, Volume *20*

Supplemental information

M2 macrophage-derived exosomal microRNA-155-5p promotes the immune escape of colon cancer by downregulating ZC3H12B

Yu-Shui Ma, Ting-Miao Wu, Chang-Chun Ling, Fei Yu, Jie Zhang, Ping-Sheng Cao, Li-Peng Gu, Hui-Ming Wang, Hong Xu, Liu Li, Zhi-Jun Wu, Gao-Ren Wang, Wen Li, Qin-Lu Lin, Ji-Bin Liu, and Da Fu

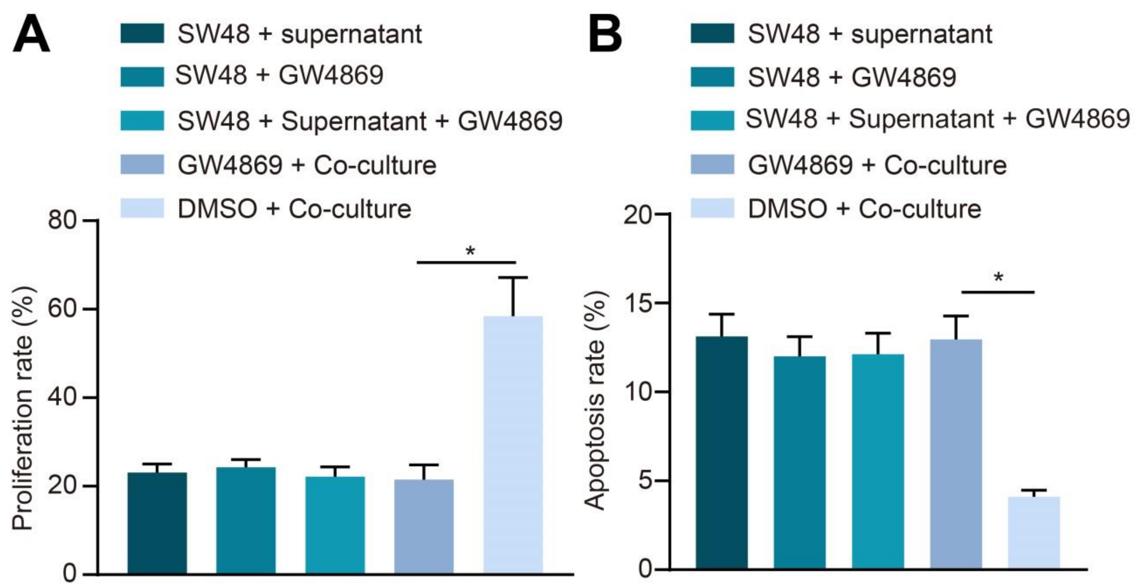

Figure S1. Inhibition of M2 macrophage-derived exosomes attenuated the proliferation and anti-apoptosis of SW48 cells. (A) EdU assay of SW48 cells proliferation after co-culture with M2 macrophages treated with GW4869. (B) Flow cytometric analysis of SW48 cell apoptosis after co-culture with M2 macrophages treated with GW4869.

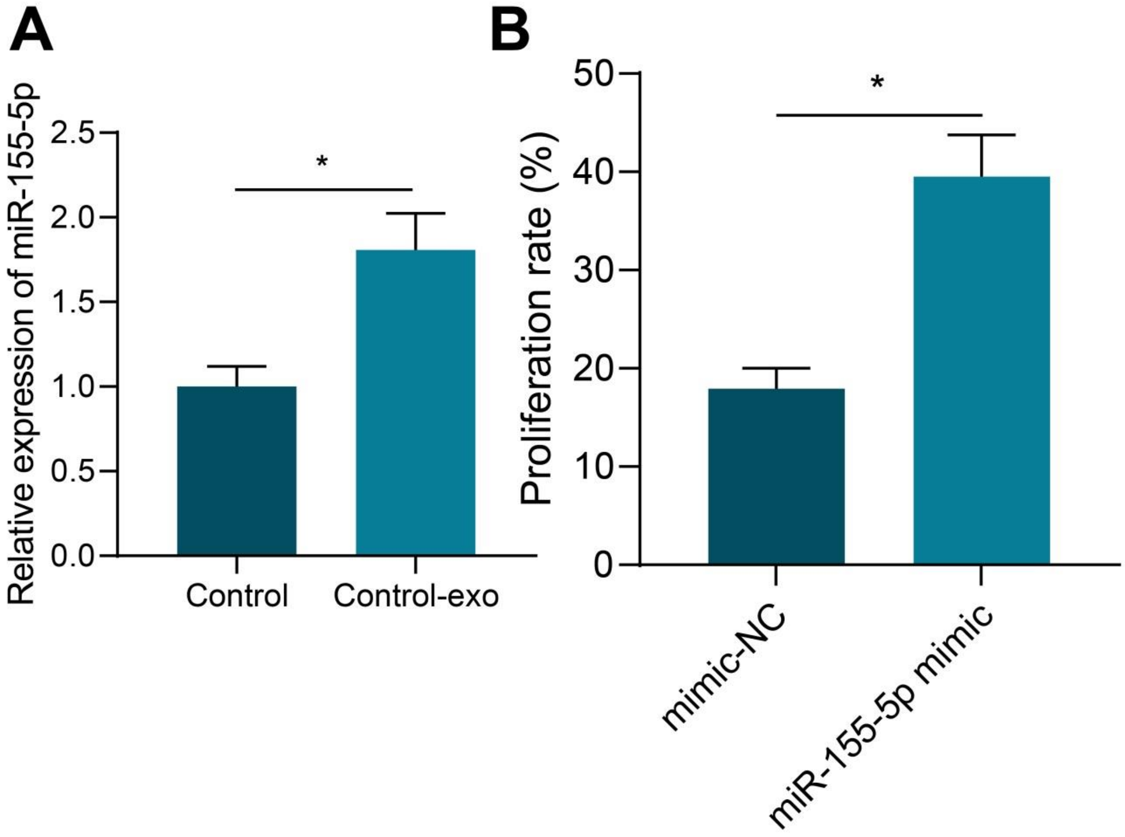

Figure S2. miR-155-5p augmented the proliferation of SW48 cells. (A) miR-155-5p expression determined by RT-qPCR in the receptor cells treated with exosomes isolated from untransfected M2 macrophages. (B) The proliferation of SW48 cells in response to miR-155-5p mimic determined by EdU staining.

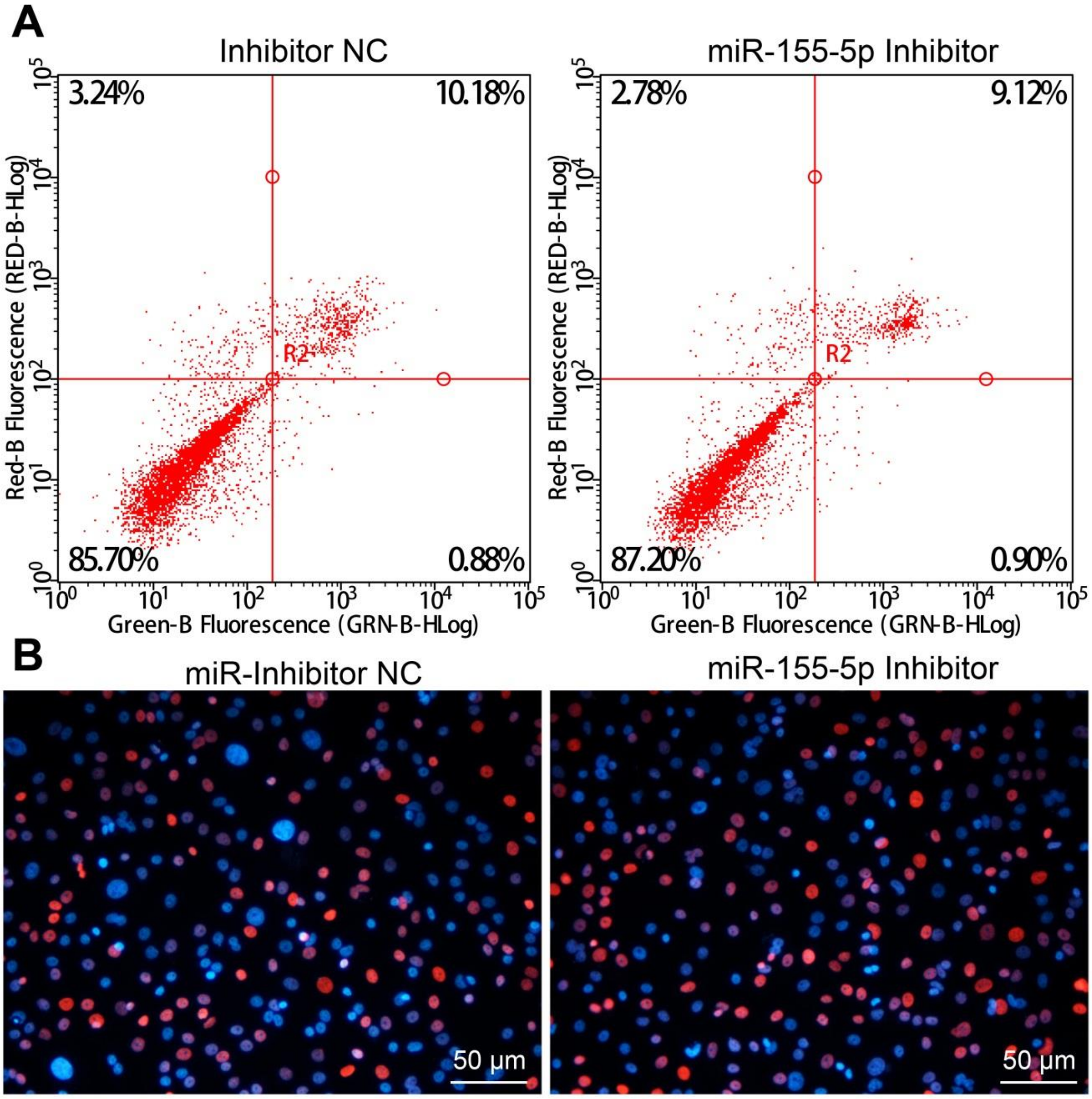

Figure S3. The proliferation and apoptosis of SW48 cells after inhibition of miR-155-5p. (A) The apoptosis of SW48 cells in response to exo-inhibitor NC or exo-miR-155-5p inhibitor determined by flow cytometry. (B) The proliferation of SW48 cells in response to exo-inhibitor NC or exo-miR-155-5p inhibitor determined by EdU staining.

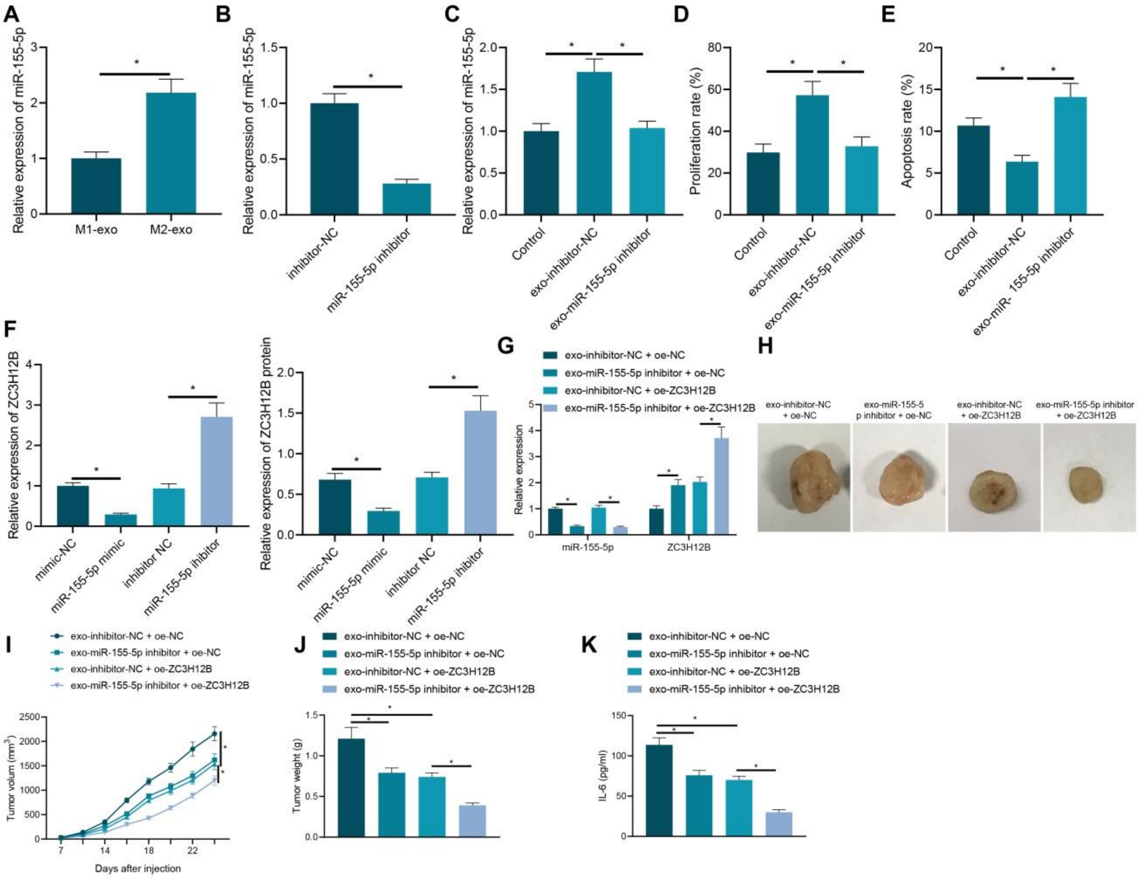

Figure S4. M2 macrophage-derived exosomal miR-155-5p promoted proliferation and repressed apoptosis of HT29 cells, as well as inhibiting immune escape *in vivo*. (A) miR-155-5p expression patterns determined by RT-qPCR in the exosomes from M1 and M2 macrophages. (B) miR-155-5p expression patterns determined by RT-qPCR in the exosomes from M2 macrophages transfected with miR-155-5p inhibitor. (C) miR-155-5p expression patterns determined by RT-qPCR in the HT29 cells co-cultured with the exosomes from M2 macrophages and transfected with miR-155-5p inhibitor. (D) HT29 cell proliferation measured by EdU assay upon treatment with exo-miR-155-5p inhibitor. (E) HT29 cell apoptosis measured by flow cytometry upon treatment with exo-miR-155-5p inhibitor. (F) ZC3H12B mRNA and protein expression patterns determined by RT-qPCR and Western blot analysis in SW48 cells following varied treatments. (G) RT-qPCR examining the expression patterns of miR-155-5p and ZC3H12B mRNA in tumor tissues from mice following varied treatments. (H) Representative images of tumors from mice following varied treatments. (I)

Tumor volume of mice. (J) Tumor weight of mice. (K) ELISA examining the expression patterns of IL-6 in spleen cell lysates of mice. The data were measurement data, and expressed as mean ± standard deviation. Comparisons between two groups were analyzed by independent sample *t* test. Comparisons among multiple groups were analyzed by one-way ANOVA, followed by Tukey's post hoc test. * $p < 0.05$. n = 5.

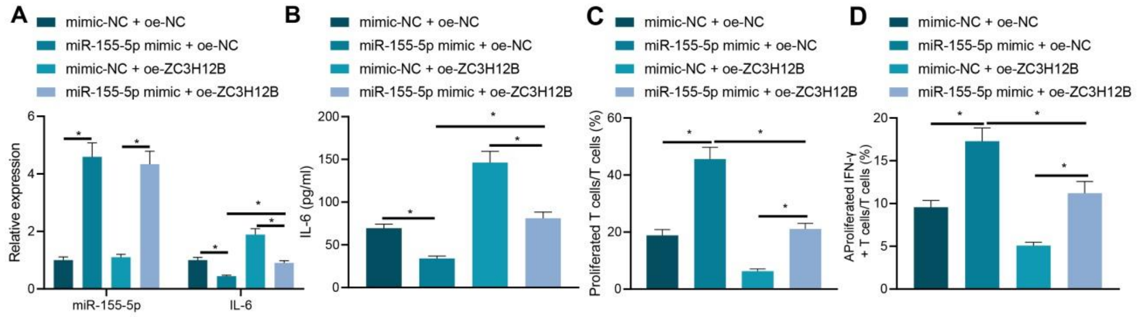

Figure S5. miR-155-5p functioned through IL-6 to regulate the ZC3H12B expression. (A) Expression patterns of miR-155-5p and IL-6 mRNA determined by RT-qPCR in the SW48 cells transfected with miR-155-5p mimic, oe-ZC3H12B or both. (B) IL-6 expression patterns determined by ELISA in the supernatants of SW48 cells transfected with miR-155-5p mimic, oe-ZC3H12B or both. (C) Proliferation of $CD3^+$ T cells determined by flow cytometry upon co-culture with SW48 cells transfected with miR-155-5p mimic, oe-ZC3H12B or both. (D) Proportion of IFN-γ^+ T cells determined by flow cytometry upon co-culture with SW48 cells transfected with miR-155-5p mimic, oe-ZC3H12B or both.

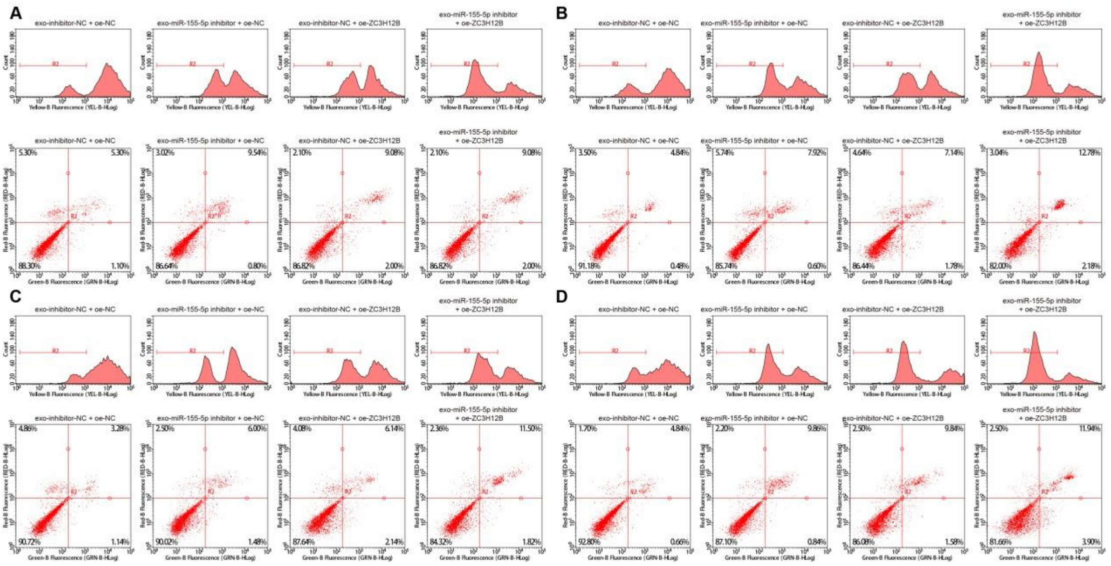

Figure S6. Measurement of expression patterns of T cell markers CD4 and CD8, M1 type markers CD14 and CD86, and M2 type markers CD163 and CD206 in the mouse tumor tissue. (A) CD4 expression patterns in mouse spleen tissues. (B) CD8 expression patterns in mouse spleen tissues examined by RT-qPCR. (C) CD14 and CD86 expression patterns in mouse spleen tissues examined by RT-qPCR. (D) CD163 and CD206 expression patterns in mouse spleen tissues examined by RT-qPCR.

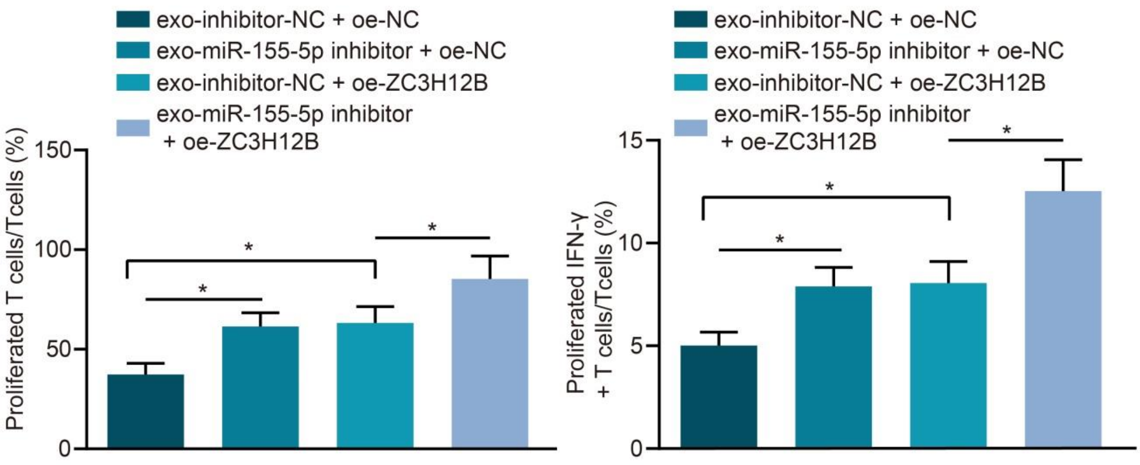

Figure S7. Flow cytometric analysis of the proportion of proliferated T cells and INF-γ^{+} T cells in spleen cells of mice treated with exo-miR-155-5p inhibitor, oe-ZC3H12B or both.

Table S1 Primer sequences for RT-qPCR

Target	Primer sequence
miR-155-5p	F: 5'-TTAATGCTAATCGTGATAGGG-3'<br>R: 5'-GTGCAGGTCCGAGGT-3'
ZC3H12B	F: 5'-CCTCGTGGGCCCAGCTCCAG-3'<br>R: 5'-TCCCATGGCTCATTGCCACATTACT-3'
IL-6	F: 5'-GACAGCCACTCACCTCTTCA-3'<br>R: 5'-AGTGCCTCTTTGCTGCTTTC-3'
U6	F: 5'-CTCGCTTCGGCAGCACA-3'<br>R: 5'-AACGCTTCACGAATTTGCGT-3'
GAPDH	F: 5'-GTCAACGGATTTGGTCTGTATT-3'<br>R: 5'-CGCUUCACGAAUUUGCGUGUCAU-3'

Note: RT-qPCR, reverse-transcription quantitative polymerase chain reaction; miR-155-5p, microRNA-155-5p; IL-6, interleukin 6; GAPDH, glyceraldehyde-3-phosphate dehydrogenase; F, forward; R, reverse.
