# Supplementary material for: M2 macrophage-derived exosomal microRNA-155-5p promotes the immune escape of colon cancer by downregulating ZC3H12B
Source: Mol Ther Oncolytics. 2021 Feb 6;20:484–98. doi: 10.1016/j.omto.2021.02.005 (PMC7932913; doi:10.1016/j.omto.2021.02.005)
Supplement: Document 2. Article plus Supplemental information [file mmc2.pdf]

Molecular Therapy
Oncolytics
Original Article

M2 macrophage-derived exosomal microRNA-155-5p promotes the immune escape of colon cancer by downregulating ZC3H12B

Yu-Shui Ma,[1,2,3,4,10] Ting-Miao Wu,[3,10] Chang-Chun Ling,[5,10] Fei Yu,[6,10] Jie Zhang,[7] Ping-Sheng Cao,[1] Li-Peng Gu,[1] Hui-Ming Wang,[2] Hong Xu,[8] Liu Li,[1] Zhi-Jun Wu,[9] Gao-Ren Wang,[9] Wen Li,[4] Qin-Lu Lin,[4] Ji-Bin Liu,[2] and Da Fu[1,3,4]

[1]Central Laboratory for Medical Research, Shanghai Tenth People's Hospital, Tongji University School of Medicine, Shanghai 200072, P.R. China; [2]Cancer Institute, Nantong Tumor Hospital, Nantong 226631, P.R. China; [3]Department of Radiology, The Fourth Affiliated Hospital of Anhui Medical University, Hefei 230012, P.R. China; [4]National Engineering Laboratory for Rice and By-product Deep Processing, College of Food Science and Engineering, Central South University of Forestry and Technology, Changsha 410004, P.R. China; [5]Department of General Surgery, The Affiliated Hospital of Nantong University, Nantong 226001, P.R. China; [6]Department of Nuclear Medicine, Shanghai Tenth People's Hospital, Tongji University School of Medicine, Shanghai 200072, P.R. China; [7]School of Medicine, Nantong University, Nantong 226019, P.R. China; [8]Department of Gastroenterology and Hepatology, Hangzhou Red Cross Hospital, Hangzhou 310003, P.R. China; [9]Department of Radiotherapy, Nantong Tumor Hospital, Nantong 226631, P.R. China

Previous evidence has highlighted M2 macrophage regulation of cancer cells via exosome shuttling of microRNAs (miRNAs or miRs). The current study set out to explore the possible role of M2 macrophage-derived exosomal miR-155-5p in regard to immune escape of colon cancer cells. Experimental data from quantitative reverse-transcriptase PCR (qRT-PCR) and western blot analysis revealed highly expressed miR-155-5p and interleukin (IL)-6 and poorly expressed ZC3H12B in M2 macrophage-derived exosomes. Additionally, miR-155-5p could be transferred by M2 macrophage-isolated exosomes to colon cancer cells, which targeted ZC3H12B by binding to the 3¿ UTR, as identified by dual luciferase reporter gene. Meanwhile, gain- and loss-of function experimentation on miR-155-5p and ZC3H12B in SW48 and HT29 cells cocultured with M2 macrophage-secreted exosomes demonstrated that miR-155-5p overexpression or ZC3H12B silencing promoted the proliferation and antiapoptosis ability of SW48 and HT29 cells, as well as augmenting the CD3$^+$ T cell proliferation and the proportion of interferon (IFN)-γ^+ T cells. Xenograft models confirmed that M2 macrophage-derived exosomal miR-155-5p reduced the ZC3H12B expression to upregulate IL-6, which consequently induced immune escape and tumor formation. Collectively, our findings indicated that M2 macrophage-derived exosomal miR-155-5p can potentially promote the immune escape of colon cancer by impairing ZC3H12B-mediated IL-6 stability reduction, thereby promoting the occurrence and development of colon cancer.

INTRODUCTION

Colon cancer is the third leading cause of cancer-related deaths across the world, with a measly survival rate of 10% if diagnosed at late stage.[1] In addition, colon cancer is highly prevalent in elderly populations, which is a major public health concern for every nation.[2] The survival rate of colon cancer is closely related to the location, stage, and size of the tumor.[3] Currently, patients suffering from stage I–III colon cancer are treated with colectomy with lymphadenectomy, while adjuvant chemotherapy is the norm for patients at stage III.[4] Unfortunately, metastasis is the leading cause of death in patients with late-stage colon cancer.[5,6] On the other hand, the process of immune escape is significantly relevant to tumor survival; thus investigating its mechanisms could aid the development of newer and more effective therapeutic options for colon cancer.[7,8]

Macrophages play critical roles in numerous biological processes, which polarize a proinflammatory (M1) or anti-inflammatory (M2) phenotype in response to environmental signals.[9] In addition, the M2 macrophage has been previously associated with colon cancer. For instance, the M2 macrophage was recently highlighted to be implicated in the progression of colon cancer.[10] Tumor-associated macrophages of the M2 phenotype have also been reported to promote the migration and metastasis of colon cancer cells.[10] Meanwhile, exosomes are known as small membrane-enclosed vesicles that function as mediators of intercellular communication.[11] More importantly, M2 macrophage-derived

Received 27 August 2020; accepted 2 February 2021;
https://doi.org/10.1016/j.omto.2021.02.005.

[10]These authors contributed equally

Correspondence: Da Fu, Central Laboratory for Medical Research, Shanghai Tenth People's Hospital, Tongji University School of Medicine, Shanghai 200072, P.R. China.
E-mail: dfau1988@126.com
Correspondence: Ji-Bin Liu, Cancer Institute, Nantong Tumor Hospital, Nantong 226631, P.R. China.
E-mail: liujibin72@yeah.net
Correspondence: Qin-Lu Lin, National Engineering Laboratory for Rice and By-product Deep Processing, College of Food Science and Engineering, Central South University of Forestry and Technology, Changsha 410004, P.R. China.
E-mail: linqinlu@126.com

 © 2021 The Authors.
This is an open access article under the CC BY license (http://creativecommons.org/licenses/by/4.0/).

exosomes have been shown to stimulate the migration and invasion of colorectal cancer cells.[12] Furthermore, macrophage-derived microRNA (miR)-155-containing exosomes can regulate the bioproperties of fibroblasts during cardiac injury.[13] Moreover, another study found that M2 macrophage-derived exosomes transferred miR-21-5p and miR-155-5p to colorectal cancer cells, leading to the promotion of cell migration and invasion.[12] Also, miR-21 shuttled by exosomes derived from M2 macrophages can further confer cisplatin resistance to gastric cancer cells.[14] This previous research highlighted that M2 macrophage-derived miR-155-5p exosomes may play a key role in colon cancer, which should be investigated in future studies.

microRNAs (miRNAs) have emerged as diagnostic and therapeutic targets for various types of cancer.[15] Colon cancer might not be an exception to this, as researchers have uncovered that miRNAs also play vital roles in colon cancer.[16,17] In addition, recent evidence illustrated that miR-155-5p is closely related to the development of colorectal cancer,[18] while miR-155-5p mediated by AU-rich elements could positively regulate the migration of colon cancer cells.[19] Another study also revealed that overexpressed miR-155-5p was involved in the progression of colon cancer.[20] Meanwhile, Zinc-finger-type-containing 12B (ZC3H12B), a new active member of the ZC3H12 protein family, is known to be involved in inflammatory processes, while the biological role of ZC3H12B depends on an intact Nedd4-BP1, YacP nucleases (NYN)/PilT N-terminal (PIN) RNase domain that contains all residues important for RNA binding and cleavage.[21] Studies have further shown that the ZC3H12B protein is implicated in the degradation of cytoplasmic viral and/or unspliced transcripts by binding to motifs resembling the splice donor sequence.[22] Additionally, an online website indicated the presence of targeted binding sites between miR-155-5p and ZC3H12B. Moreover, ZC3H12B can also bind to the interleukin (IL)-6 mRNA *in vivo*, regulate its turnover, and consequently lead to reduced IL-6 protein expression upon stimulation with IL-1β.[21] The aforementioned discussion triggered the hypothesis that M2 macrophage-derived exosomal miR-155-5p might mediate the progression of colon cancer via ZC3H12B. As a result, the current study set out to explore whether M2 macrophage-derived exosomal miR-155-5p regulates immune escape in colon cancer and the underlying mechanisms involving ZC3H12B.

RESULTS

M2 macrophage-derived exosomes affected colon cancer progression

M2 macrophages were obtained from tissues isolated from clinically diagnosed colon cancer patients. Subsequent immunostaining results (Figure 1A) illustrated that the samples were positive for M2 macrophage markers CD68, CD163, and CD206. In addition, findings from western blot analysis (Figure 1B) revealed the presence of increased protein expression levels of CD68, CD163, and CD206 after isolation, both indicating the successful isolation of M2 macrophage. The isolated M2 macrophages were then cocultured with SW48 colon cancer cells, in order to investigate whether M2 macrophages affected colon cancer progression. After 48 h of culture, the SW48 cells were isolated and underwent 5-ethynyl-2¿-deoxyuridine (EdU) assay and flow cytometry to examine apoptosis and proliferation in the cells. The results (Figures 1C and 1D) showed that compared with SW48 cells cocultured with M1 macrophages or phosphate-buffered saline (PBS), those cocultured with M2 macrophages presented with increased proliferation and antiapoptotic abilities, indicating that M2 macrophages were positively correlated with colon cancer development.

To further explore whether M2 macrophages transferred exosomes to colon cancer cells, influencing cell proliferation and apoptosis, nanoparticle tracking analysis was applied to analyze the exosome size and distribution. The results (Figure 1E) demonstrated that the exosome particle size was primarily ~100 nm. In addition, observation under a transmission electron microscope (Figure 1F) illustrated that the secretions presented with typical morphological features of exosomes and appeared to have a spherical structure formed by a lipid bilayer molecular membrane. Furthermore, western blot analysis results (Figure 1G) showed that exosomes produced by M2 macrophages were found to express the specific marker proteins CD81 and CD63. The above results indicated that the isolated exosomes were indeed secreted by M2 macrophages.

Additionally, the exosomes were labeled with 3'-dioctadecyloxacarbocyanine perchlorate (DiO) to investigate whether colon cancer cells internalized the exosomes secreted by M2 macrophages. After 48 h of coculture of exosomes with SW48 cells, green fluorescence was observed in SW48 cells (Figure 1H), which revealed that colon cancer cells could take up the exosomes derived from M2 macrophages. Subsequently, M2 macrophages were treated with the exosome inhibitor GW4869 to inhibit the release of exosomes, and the supernatant of M2 macrophages was then cocultured with SW48 cells. The results of the EdU assay demonstrated a decline in SW48 cell proliferation following coculture with GW4869-treated M2 macrophages (Figure 1I). Additionally, the protein expression levels of exosome-specific marker proteins CD63 and CD81 were found to be diminished in SW48 cells cocultured with GW4869-treated M2 macrophages (Figure 1J). Meanwhile, as shown by the results of EdU assay and flow cytometry, no significant differences were noted in regard to the cell proliferation and antiapoptosis abilities of SW48 cells after coculture with the supernatants of M2 macrophages or those treated with GW4869 alone or in combination with dimethyl sulfoxide (DMSO). On the other hand, SW48 cells cocultured with the supernatants of M2 macrophages treated with GW4869 presented with decreased cell proliferation (Figure S1A) and antiapoptosis ability (Figure S1B). Altogether, the abovementioned data indicated that the M2 macrophage-derived exosomes influenced the biological characteristics of colon cancer cells in promoting proliferation and antiapoptosis upon internalization by colon cancer cells and subsequently manipulated the occurrence and development of colon cancer.

M2 macrophage-derived exosomal miR-155-5p promoted colon cancer cell proliferation and antiapoptosis

After uncovering that M2 macrophage-derived exosomes did confer an effect on colon cancer, we performed quantitative reverse-transcription polymerase chain reaction (qRT-PCR) to examine the expression patterns of miR-155-5p in M2 macrophage-derived

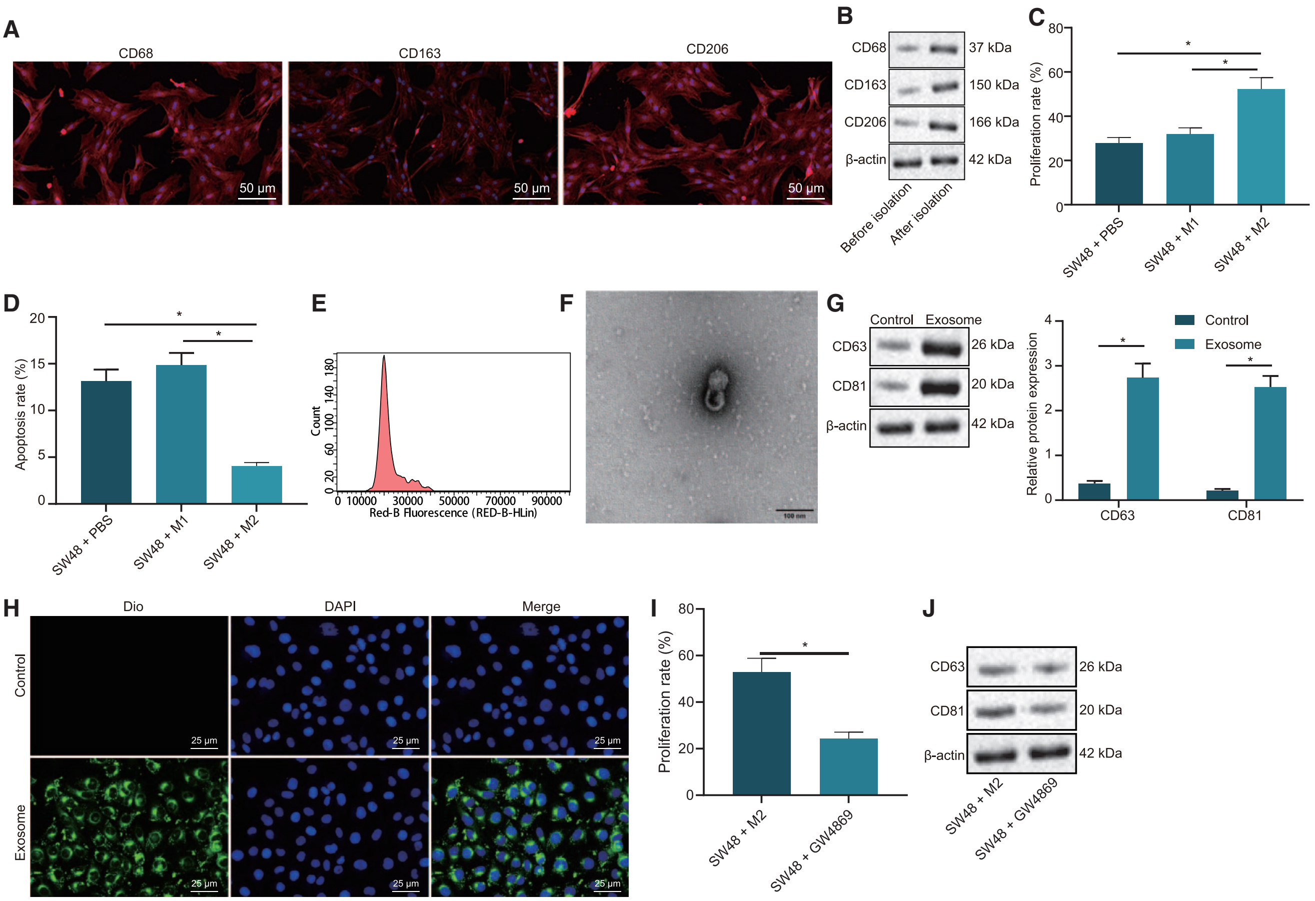

Figure 1. Colon cancer progression was affected by M2 macrophage-derived exosomes

(A) Immunostaining of expression of M2 macrophage markers (CD68, CD163, and CD206) (×200). (B) Western blot analysis of protein expression patterns of the M2 macrophage markers (CD68, CD163, and CD206). (C) The effect of M2 macrophages on SW48 cell proliferation detected by EdU assay. (D) The effect of M2 macrophages on SW48 cell apoptosis rate measured by flow cytometry. (E) Nanoparticle tracking analysis of particle size of exosomes. (F) The ultrastructure of the exosomes (×5,000) observed under a transmission electron microscope. (G) Western blot analysis of the expression of exosome markers (CD63 and CD81). Control, the supernatant after exosome isolation. Exosome, extracted exosomes. (H) Internalization of M2 macrophage-derived exosomes in SW48 cells (×400). (I) SW48 cell proliferation measured by EdU assay upon coculture with GW4869-treated M2 macrophages. (J) Western blot analysis of exosome-specific marker proteins CD63 and CD81 in SW48 cells cocultured with GW4869-treated M2 macrophages. The data are all measurement data expressed as mean ± standard deviation and compared by independent-sample t test. *$p < 0.05$. The cell experiment was repeated three times.

exosomes from SW48 cells, to further explore the effect of M2 macrophage-derived exosomal miR-155-5p on colon cancer progression. As depicted in Figure 2A, miR-155-5p was found to be highly expressed in M2 macrophage-derived exosomes. Subsequently, M2 macrophages were transfected with Cy3-labeled miR-155-5p and cocultured with SW48 cells. After 48 h of culture, the percentage of Cy3-positive cells in SW48 cells was examined by means of immunofluorescence and flow cytometry (Figure 2B), which revealed that Cy3-positive cells presented with an evident enhancement in number in SW48 cells, indicating that miR-155-5p was transferred from M2 macrophages to colon cancer cells via exosomes.

To verify whether M2 macrophage-derived exosomes play an oncogenic role in colon cancer by miR-155-5p, a miR-155-5p inhibitor was introduced into the M2 macrophages. Subsequent findings from qRT-PCR (Figure 2C) demonstrated that miR-155-5p expression levels were decreased in exosomes after treatment with miR-155-5p inhibitor in M2 macrophages. After colon cancer cells were cocultured with exosomes, miR-155-5p expression patterns in SW48 cells were reexamined. As illustrated in Figure 2D, SW48 cells presented with diminished miR-155-5p expression levels in SW48 cells cocultured with exosomes derived from miR-155-5p inhibitor-treated M2 macrophages (exo-miR-155-5p inhibitor) relative to those treated with exo-inhibitor negative control (NC). Additionally, proliferation and apoptosis abilities were examined in the SW48 cells, and it was found that coculture with exo-miR-155-5p inhibitor led to reduced cell proliferation (Figure 2E) and elevated cell apoptosis (Figure 2F) of SW48 cells. Furthermore, we used the exosomes isolated

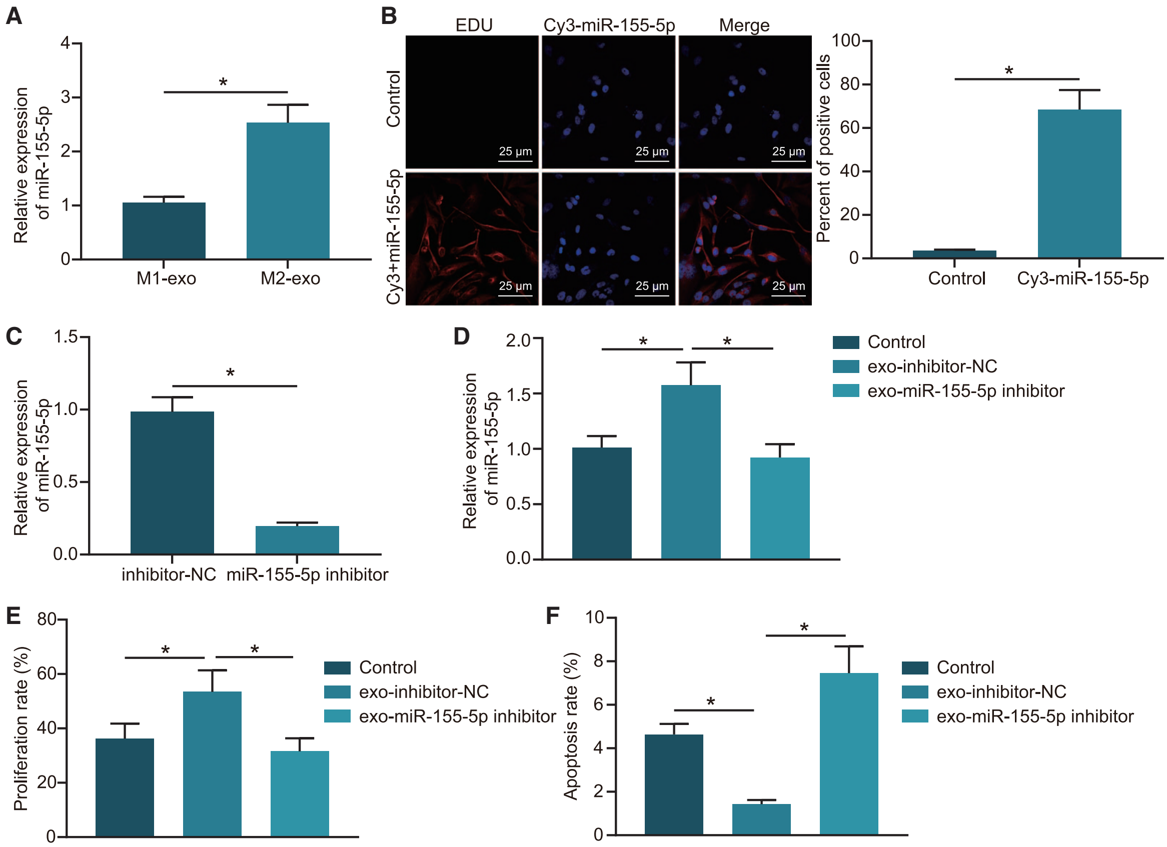

Figure 2. M2 macrophage-derived exosomes transferred miR-155-5p to promote proliferation and to repress apoptosis of SW48 cells

(A) qRT-PCR examining the expression patterns of miR-155-5p in M2 macrophage-derived exosomes compared with M1 macrophage-derived exosomes (normalized to U6). (B) Immunofluorescence demonstrating transferring of miR-155-5p from M2 macrophages to SW48 cells (scale bars, 25 μm). (C) qRT-PCR examining the expression patterns of miR-155-5p in exo-miR-155-5p inhibitor and exo-inhibitor NC (normalized to U6). (D) qRT-PCR analysis of miR-155-5p in SW48 cells after coculture with exo-miR-155-5p inhibitor and exo-inhibitor NC (normalized to U6). (E) SW48 cell proliferation after coculture with exo-miR-155-5p inhibitor and exo-inhibitor NC detected by EdU assay. (F) SW48 cell apoptosis after coculture with exo-miR-155-5p inhibitor and exo-inhibitor NC detected by flow cytometry. The data are measurement data expressed as mean ± standard deviation. Comparisons between two groups were analyzed by independent-sample t test. Comparisons among multiple groups were analyzed by one-way ANOVA, followed by Tukey's post hoc test. *p < 0.05. The cell experiment was repeated three times.

from untransfected M2 macrophages to treat the receptor cells and detected the expression patterns of miR-155-5p in the receptor cells. The results demonstrated an enhancement in miR-155-5p expression levels in the receptor cells (Figure S2A). Also, miR-155-5p was overexpressed in the SW48 cells, and EdU assay revealed that proliferation was enhanced in the SW48 cells (Figure S2B), while the proliferation and apoptosis abilities of SW48 cells remained unchanged in response to exo-miR-155-5p-inhibition (Figures S3A and S3B). These results suggested that miR-155-5p could indeed affect the apoptosis and proliferation of SW48 cells. However, miR-155-5p was rarely expressed in SW48 cells, and miR-155-5p inhibition did not confer a significant effect on the apoptosis and proliferation abilities of SW48 cells.

The aforementioned experiments were repeated in another colon cancer cell line, HT29, and similar results were documented. It was identified that miR-155-5p was highly expressed in the exosomes derived from M2 macrophages (Figure S4A). Moreover, upregulated expression levels of miR-155-5p were detected in the exosomes from M2 macrophages transfected with miR-155-5p inhibitor by qRT-PCR (Figure S4B) while being diminished in the HT29 cells cocultured with the exosomes from M2 macrophages transfected with miR-155-5p inhibitor (Figure S4C). In addition, the results of EdU assay and flow cytometry revealed an increase in HT29 cell proliferation (Figure S4D) and a decline in HT29 cell apoptosis (Figure S4E) in the presence of exo-inhibitor-NC, while exo-miR-155-5p-inhibitor abolished the effect of exo-inhibitor-NC on the aforementioned factors. Collectively, these results suggested that downregulation of exosomal miR-155-5p could reduce the cancer-promoting effect of M2 macrophage-derived exosomes on colon cancer cells.

ZC3H12B was a target gene of miR-155-5p

Furthermore, we investigated the downstream mechanism of M2 macrophage-derived exosomal miR-155-5p in colon cancer progression. First, the TargetScan website (http://www.targetscan.

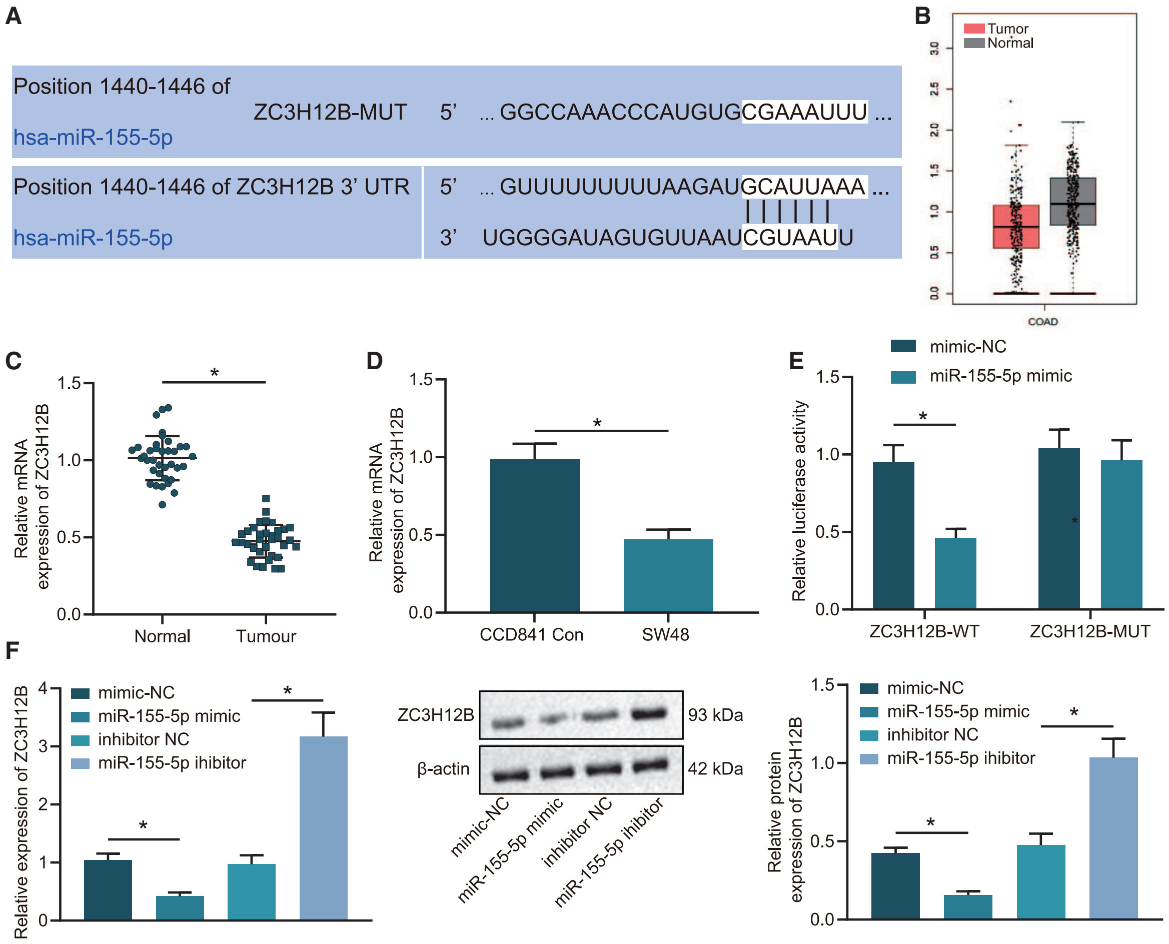

Figure 3. miR-155-5p negatively targeted ZC3H12B

(A) The online site prediction of the binding site of miR-155-5p to ZC3H12B. (B) GEPIA website prediction of ZC3H12B expression patterns in colon cancer. (C) qRT-PCR examining the mRNA expression patterns of ZC3H12B in colon cancer and adjacent normal tissue samples (n = 36; normalized to GAPDH). (D) qRT-PCR examining the mRNA expression patterns of ZC3H12B in SW48 colon cancer cells and CCD841CoN human normal colon epithelial cells (normalized to GAPDH). (E) Dual luciferase reporter assay verifying the targeted binding of miR-155-5p to ZC3H12B. (F) qRT-PCR and western blot analysis examining the mRNA and protein expression patterns of ZC3H12B in SW48 cells after alteration of miR-155-5p (normalized to GAPDH). The data are measurement data expressed as mean ± standard deviation. Comparisons between two groups were analyzed by unpaired t test. Data were compared between cancer tissues and adjacent normal tissues by paired t test. Comparisons among multiple groups were analyzed by one-way ANOVA, followed by Tukey's post hoc test. *$p < 0.05$. The cell experiment was repeated three times.

org/vert_71/) (Figure 3A) predicted the presence of a targeted binding relationship between ZC3H12B and miR-155-5p. In addition, the GEPIA website (http://gepia.cancer-pku.cn/detail.php?gene=ZC3H12B) indicated that ZC3H12B was underexpressed in colon cancer (Figure 3B). Consequently, we speculated that miR-155-5p may play a role in colon cancer via ZC3H12B regulation.

Subsequently, qRT-PCR was performed to determine the mRNA expression patterns of ZC3H12B in colon cancer tumor and adjacent normal tissues (Figure 3C) and SW48 colon cancer cells and CCD841CoN human normal colon epithelial cells (Figure 3D). It was found that ZC3H12B was poorly expressed in colon cancer tumor tissues and cells. In addition, the results of dual luciferase reporter gene assay (Figure 3E) showed that the luciferase activity of ZC3H12B 3¿ untranslated region (3¿ UTR) wild type (WT) was decreased by miR-155-5p mimic, while no significant differences were detected in the luciferase activity of ZC3H12B 3¿ UTR mutant type (MUT) ($p > 0.05$), indicating that miR-155-5p could specifically bind to ZC3H12B. SW48 cells were further transfected with miR-155-5p mimic or miR-155-5p inhibitor, and findings from qRT-PCR and western blot analysis (Figure 3F) showed that ZC3H12B expression levels were decreased in miR-155-5p mimic-treated SW48 cells and enhanced in the SW48 cells treated with miR-155-5p inhibitor. The qRT-PCR and western blot experiments were also repeated in the HT29 cell line, which also revealed reduced ZC3H12B mRNA and protein expressions in the HT29 cells after miR-155-5p mimic treatment and elevated expressions after miR-155-5p inhibitor treatment (Figure S4F). Collectively, these

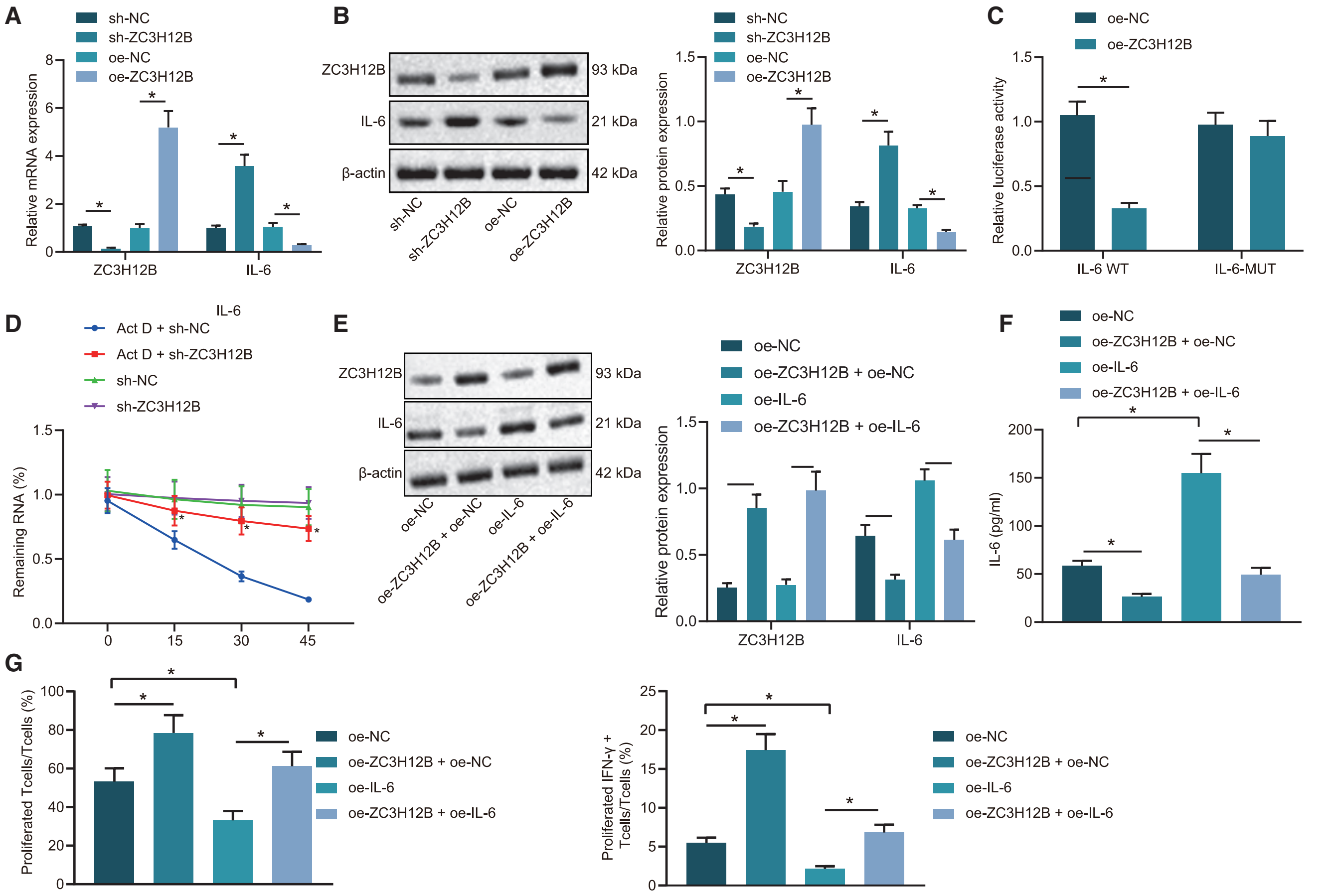

Figure 4. ZC3H12B downregulated IL-6 expression to repress immune escape

(A) ZC3H12B and IL-6 mRNA expression patterns after alteration of ZC3H12B detected by qRT-PCR. (B) ZC3H12B and IL-6 protein expression patterns after alteration of ZC3H12B detected by western blot analysis. (C) Luciferase reporter gene system validating the binding of ZC3H12B and IL-6 mRNA 3¿ UTR. (D) The residual amount of IL-6 mRNA at different time points in SW48 cells treated with sh-ZC3H12B and actinomycin D (Act D) measured by qRT-PCR (normalized to GAPDH). (E) Western blot analysis of ZC3H12B and IL-6 protein expression patterns in SW48 cells after overexpression of ZC3H12B and IL-6 (normalized to GAPDH). (F) ELISA examining IL-6 expression patterns in the supernatant of SW48 cells after overexpression of ZC3H12B and IL-6. (G) Flow cytometry examining the proliferation of $CD3^+$ T cells and the proportion of activated IFN-γ^+ T cells after coculture of SW48 colon cancer cells treated with oe-ZC3H12B and oe-IL-6. The data are measurement data expressed as mean ± standard deviation. Comparisons between two groups were analyzed by independent-sample t test. Comparisons among multiple groups were analyzed by one-way ANOVA, followed by Tukey's post hoc test. *$p < 0.05$. #$p < 0.05$. The cell experiment was repeated three times.

results indicated that ZC3H12B expression was negatively targeted by miR-155-5p.

ZC3H12B suppressed immune escape through IL-6 downregulation

Existing literature indicates that the interaction of ZC3H12B with the stem-loop structure in the IL-6 3¿ UTR can regulate the stability of IL-6 mRNA.[21] Based on this data, we speculated whether ZC3H12B affected the development of colon cancer by regulating IL-6. To confirm this speculation, ZC3H12B expression was silenced and overexpressed in colon cancer cells. ZC3H12B and IL-6 mRNA and protein expression patterns were subsequently examined by qRT-PCR (Figure 4A) and western blot analysis (Figure 4B), and it was found that ZC3H12B expression levels were elevated and those of IL-6 were decreased after treatment with overexpression (oe)-ZC3H12B, which could be reversed after the silencing of ZC3H12B. Meanwhile, the results of dual luciferase reporter assay showed that oe-ZC3H12B treatment reduced the luciferase activity in pmirGLO_IL-6 3¿-UTR reporter plasmid (Figure 4C), which confirmed the existence of a binding relationship between ZC3H12B and IL-6 mRNA 3¿ UTR. Furthermore, to investigate whether ZC3H12B negatively regulated IL-6 by affecting its mRNA stability, ZC3H12B expression was silenced in SW48 cells, which were then treated with actinomycin D. The mRNA expression patterns of IL-6 were measured at different time points, and it was found that the residual amount of IL-6 mRNA after short hairpin RNA (sh)-ZC3H12B treatment increased at each time point (Figure 4D), indicating that downregulation of ZC3H12B could reduce the degradation rate of IL-6 mRNA. Together, the abovementioned results suggested that ZC3H12B downregulated IL-6 by decreasing the stability of IL-6 mRNA.

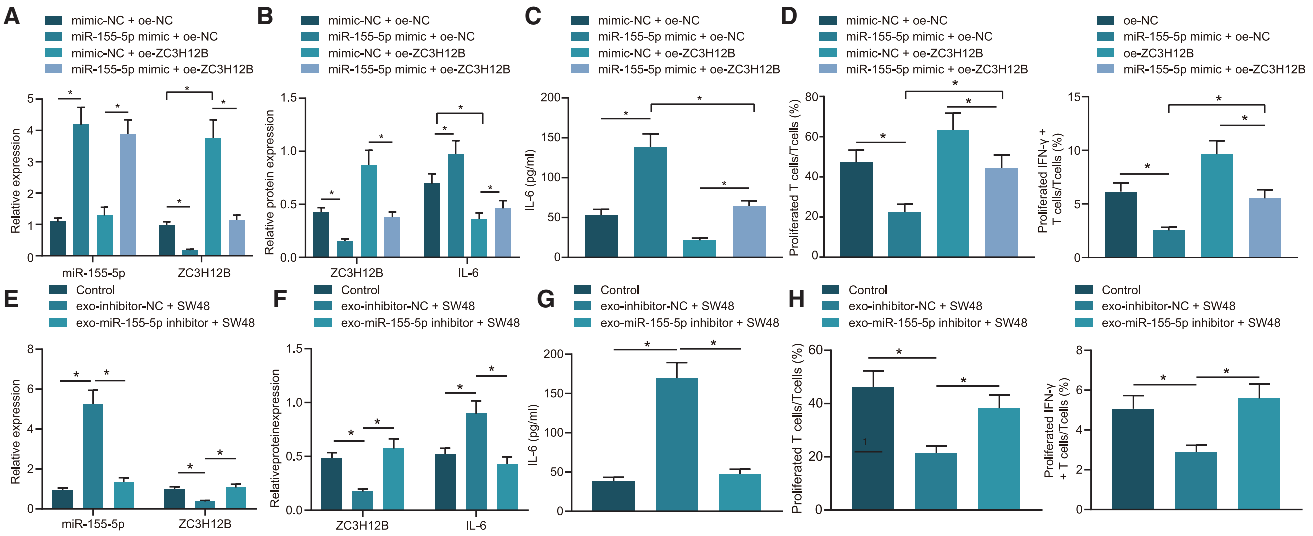

Figure 5. miR-155-5p promoted immune escape of colon cancer by downregulating the expression of ZC3H12B *in vitro*

SW48 cells were transfected with oe-NC, miR-155-5p mimic + oe-NC, oe-ZC3H12B, or miR-155-5p mimic + oe-ZC3H12B. (A) qRT-PCR examining the expression patterns of miR-155-5p expression and ZC3H12B mRNA in SW48 cells. (B) Western blot analysis examining the protein expression patterns of ZC3H12B and IL-6 in SW48 cells. (C) ELISA examining the expression patterns of IL-6 in the supernatant of SW48 cells. (D) Flow cytometry examining the proliferation of T cells and the proportion of activated INF-γ^+ T cells after coculture of SW48 cells with different transfections. (E) qRT-PCR examining the expression patterns of miR-155-5p and ZC3H12B mRNA in SW48 cells after coculture with exo-miR-155-5p inhibitor (SW48 cells were treated with PBS as control, the same as below). (F) Western blot analysis examining the expression patterns of ZC3H12B and IL-6 protein in SW48 cells after coculture with exo-miR-155-5p inhibitor. (G) ELISA examining the expression patterns of IL-6 in the supernatant of SW48 cells after coculture with exo-miR-155-5p inhibitor. (H) Flow cytometry examining the proliferation of T cells and the proportion of activated IFN-γ^+ T cells after coculture with exo-miR-155-5p inhibitor-treated SW48 cells. The data are measurement data expressed as mean ± standard deviation. Comparisons between two groups were analyzed by independent-sample t test. Comparisons among multiple groups were analyzed by one-way ANOVA, followed by Tukey's post hoc test. *$p < 0.05$. The cell experiment was repeated three times.

To verify whether ZC3H12B inhibits immune escape by downregulating IL-6 in colon cancer, ZC3H12B or IL-6 was overexpressed in SW48 cells. Subsequently, western blot analysis was conducted to assess the expression patterns of ZC3H12B and IL-6 (Figure 4E), and IL-6 content in the supernatant of SW48 cells was evaluated by means of enzyme-linked immunosorbent assay (ELISA) (Figure 4F). It was found that overexpression of ZC3H12B reduced the IL-6 expression. Compared with treatment with oe-IL-6 alone, SW48 cells treated with oe-ZC3H12B + oe-IL-6 presented with decreased IL-6 expression levels. Later, SW48 cells and CD3$^+$ T cells were cocultured, and flow cytometry was then performed to examine CD3$^+$ T cell proliferation and the proportion of interferon (IFN)-γ^+ T cells. As depicted in Figure 4G, proliferation of CD3$^+$ T cells and the proportion of IFN-γ^+ T cells were found to be increased after coculture with ZC3H12B-overexpressed SW48 cells. Meanwhile, coculture with IL-6-overexpressed SW48 cells resulted in decreased CD3$^+$ T cell proliferation and proportion of IFN-γ^+ T cells, which could be reversed by coculture with SW48 cells treated with oe-ZC3H12B + oe-IL-6. These results indicated that ZC3H12B inhibited immune escape by decreasing the IL-6 expression.

M2 macrophage-derived exosomal miR-155-5p promoted immune escape in colon cancer through ZC3H12B

Our initial results indicated that ZC3H12B affected immune escape through IL-6, but whether miR-155-5p promotes immune escape through the regulation of ZC3H12B in colon cancer remained unclear. In order to achieve clarity, we overexpressed miR-155-5p and ZC3H12B in SW48 cells, after which qRT-PCR was performed to examine the transfection efficiency (Figure 5A). In addition, western blot analysis was applied to examine the expression patterns of ZC3H12B and IL-6 in SW48 cells. It was found that IL-6 expression levels were elevated while those of ZC3H12B were reduced after treatment with miR-155-5p mimic + oe-NC in comparison with mimic-NC + oe-NC, which could be abolished by treatment with miR-155-5p mimic + oe-ZC3H12B (Figure 5B). Furthermore, the expression patterns of IL-6 in the supernatant of SW48 cells were detected by ELISA, and the results showed that IL-6 content was elevated after treatment with miR-155-5p mimic + oe-NC compared with treatment with mimic-NC + oe-NC, while the IL-6 content was also found to be elevated in response to miR-155-5p mimic + oe-ZC3H12B treatment compared with mimic-NC + oe-ZC3H12B treatment (Figure 5C).

Moreover, SW48 cells after different treatments were cocultured with CD3$^+$ T cells and tested with flow cytometry to examine the proliferation of CD3$^+$ T cells and the proportion of IFN-γ^+ T cells. It was found that proliferation of CD3$^+$ T cells and the proportion of IFN-γ^+ T cells were decreased after coculture with SW48 cells treated with miR-155-5p mimic + oe-NC, while these trends could be abrogated by coculture with SW48 cell treated with miR-155-5p mimic + oe-ZC3H12B.

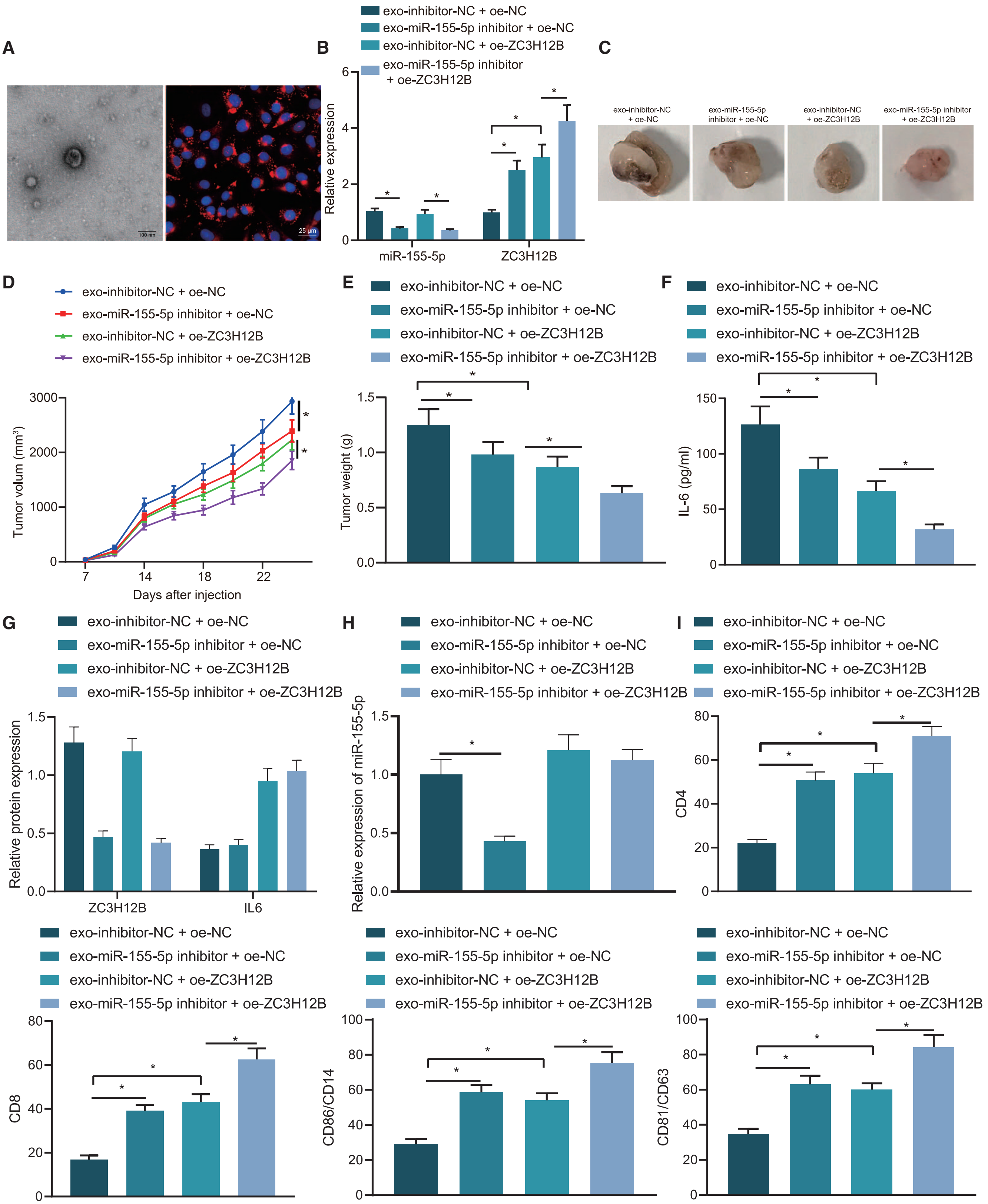

(legend on next page)

Meanwhile, the proliferation of CD3$^+$ T cells and the proportion of IFN-γ^+ T cells after coculture with SW48 cells treated with miR-155-5p mimic + oe-ZC3H12B were lower than those after coculture with oe-ZC3H12B-treated SW48 cells (Figure 5D). These results indicated that, *in vitro*, miR-155-5p promoted immune escape via ZC3H12B downregulation.

To further verify whether M2 macrophage-derived exosomal miR-155-5p can cause immune escape in colon cancer, miR-155 was overexpressed or inhibited in M2 macrophages and then cocultured with SW48 cells. qRT-PCR was applied to detect the miR-155-5p expression and ZC3H12B mRNA expression patterns in SW48 cells (Figure 5E), and western blot analysis was performed to measure the ZC3H12B and IL-6 protein expression patterns (Figure 5F). In addition, ELISA was utilized to examine the IL-6 contents in the supernatants of SW48 cells (Figure 5G). It was found that miR-155-5p and IL-6 expression levels were decreased while those of ZC3H12B were enhanced in SW48 cells after treatment with exo-miR-155-5p inhibitor. In addition, exosome-treated SW48 cells were cocultured with CD3$^+$ T cells and detected with flow cytometry to assess the proliferation of CD3$^+$ T cells and the proportion of IFN-γ^+ T cells. As illustrated in Figure 5H, the proliferation of CD3$^+$ T cells and the proportion of IFN-γ^+ T cells were found to be increased after coculture with exo-miR-155-5p inhibitor-treated SW48 cells. The results of qRT-PCR indicated that SW48 cells overexpressing miR-155-5p presented with decreased IL-6 expression levels. In comparison to simultaneous overexpression of miR-155-5p and ZC3H12B, IL-6 expression levels exhibited a more pronounced decline upon single miR-155-5p overexpression (Figure S5A). This finding suggested that miR-155-5p influenced the expression of IL-6 by regulating ZC3H12B. Meanwhile, results from ELISA demonstrated that IL-6 expression levels were decreased in the supernatants of SW48 cells after miR-155-5p overexpression. Reduced IL-6 expression levels were noted upon simultaneous overexpression of miR-155-5p and ZC3H12B compared to single ZC3H12B overexpression (Figure S5B). Flow cytometric data further revealed that the proliferation of CD3$^+$ T cells and the proportion of IFN-γ^+ T cells were increased upon coculture with SW48 cells transfected with miR-155-5p mimic or both miR-155-5p mimic and oe-ZC3H12B (Figures S5C and S5D). These experimental data confirmed that miR-155-5p functioned through IL-6 and then regulated ZC3H12B, thus affecting immune escape of colon cancer. Together, these results indicated that miR-155-5p carried by M2 macrophage-derived exosomes could promote the immune escape of colon cancer cells through the regulation of ZC3H12B.

M2 macrophage-derived exosomal miR-155-5p reduced ZC3H12B expression to promote immune escape in colon cancer *in vivo*

Finally, we determined whether the M2 macrophage-derived exosomes were successfully injected into mice. Transmission electron microscope and immunofluorescence data revealed that exosomes were expressed in mice, confirming the successful injection (Figure 6A). In order to further investigate the effect of M2 macrophage-derived exosomal miR-155-5p on immune escape in colon cancer, we treated the exosomes derived from M2 macrophages with miR-155-5p inhibitor, and ZC3H12B was overexpressed in SW48 cells treated with exo-miR-155-5p inhibitor. Subsequently, SW48 cells were coinjected into nude mice with CD3$^+$ T cells, and the corresponding exosomes were injected every 3 days. The results demonstrated that the growth rate, volume, and weight of tumors were reduced in the mice treated with exo-miR-155-5p inhibitor + oe-NC or exo-inhibitor-NC + oe-ZC3H12B and were further reduced after treatment with exo-miR-155-5p inhibitor + oe-ZC3H12B (Figures 6B–6E). The aforementioned experiments were also carried out in HT29 cells, in which treatment with exo-miR-155-5p inhibitor + oe-NC or exo-inhibitor-NC + oe-ZC3H12B resulted in decreased growth rate, volume, and weight of tumors, which were further decreased after treatment with exo-miR-155-5p inhibitor + oe-ZC3H12B (Figures S4G–S4K). These findings suggested that M2 macrophage-derived exosomal miR-155-5p promoted the development of colon cancer by inhibiting the expression of ZC3H12B *in vivo*.

Spleen belongs to the reticular skin system and is the largest lymphoid organ of the human body. The marginal zone (MZ) of the spleen is located at the junction of red pulp and white pulp, where lymphocytes are sparse, primarily comprised of B cells, but there are more macrophages. It serves as an important area of the spleen to capture and recognize antigens and induce immune responses. When the sympathetic nerve impulse is released, the expressions of immune cytokine-related genes in the spleen are altered, especially those of IL-1 and IL-6. To elucidate the mechanism of M2 macrophage-derived exosomal miR-155-5p *in vivo* affecting immune escape, we detected the expression patterns of IL-6 in spleen cell lysates by ELISA. The results showed (Figure 6F) that IL-6 expression levels were diminished in

Figure 6. miR-155-5p delivered by M2 macrophage-derived exosomes diminished ZC3H12B expression to accelerate immune escape in colon cancer *in vivo*

Mice were injected with SW48 cells treated with exo-inhibitor-NC + oe-NC, exo-miR-155-5p inhibitor + oe-NC, exo-inhibitor-NC + oe-ZC3H12B, or exo-miR-155-5p inhibitor + oe-ZC3H12B. (A) Expression patterns of M2 macrophage-derived exosomes analyzed by transmission electron microscope and immunofluorescence assay in mouse tumor tissues. (B) qRT-PCR examining the expression patterns of miR-155-5p expression and ZC3H12B mRNA in mouse tumor tissues. (C) Tumor growth of mice. (D) Tumor volume of mice. (E) Tumor weight of mice. (F) ELISA examining the expression patterns of IL-6 in spleen cell lysates of mice. (G) The expression patterns of ZC3H12B and IL-6 protein in tumor tissues of mice without any other treatments determined by immunohistochemistry. (H) miR-155-5p expression patterns in tumor tissues of mice without any other treatments. (I) Flow cytometry examining T cells in spleen cells of mice without any other treatments. The data are measurement data expressed as mean ± standard deviation. Comparisons between two groups were analyzed by independent-sample t test. Comparisons among multiple groups were analyzed by one-way ANOVA, followed by Tukey's post hoc test. *$p < 0.05$. n = 5.

mice treated with exo-miR-155-5p inhibitor + oe-NC or exo-inhibitor-NC + oe-ZC3H12B and were further decreased in mice treated with exo-miR-155-5p inhibitor + oe-ZC3H12B. We further detected the expression patterns of miR-155-5p, ZC3H12B, and IL-6 in tumor tissues of mice. At the same time, the expression patterns of ZC3H12B, T cell markers CD4 and CD8, M1 type markers CD14 and CD86, and M2 type markers CD163 and CD206 (Figures 6G–6I; Figures S6A–S6D) were also detected in mouse tumor tissues. Furthermore, the activation of T cells was examined in a spleen cell suspension from the mice by means of flow cytometry. The results documented (Figure S7) that the proportion of IFN-γ^+ T cells was enhanced in mice treated with exo-miR-155-5p inhibitor + oe-NC or exo-inhibitor-NC + oe-ZC3H12B while being further elevated in mice treated with exo-miR-155-5p inhibitor + oe-ZC3H12B. Taken together, these findings indicated that miR-155-5p transferred by M2 macrophage-derived exosomes promoted the expression of IL-6 expression through downregulation of ZC3H12B, thus inhibiting T cell immune response and promoting tumor formation.

DISCUSSION

The poor prognosis of colon cancer is associated with the development of perforation and obstruction, which are recognized as emergency complications.[23] In addition, the lack of efficient biomarkers that serve as indicators of tumor invasion accounts for the high mortality and tumor metastasis in colon cancer patients.[24,25] At present, explorations are being conducted to find predictive biomarkers for colon cancer.[26] The current study set out to investigate the role of M2 macrophage-derived exosomal miR-155-5p in colon cancer, and the findings obtained evidenced that miR-155-5p was transferred by M2 macrophage-derived exosomes to tumor cells, where it targeted ZC3H12B, implicated in the upregulation of IL-6 expression, consequently promoting immune escape of colon cancer.

First, our findings revealed that M2 macrophage-secreted exosomes could significantly promote the proliferation and antiapoptosis abilities of colon cancer cells. Consistently, previous studies have illustrated that tumor-associated macrophages serve as the primary stromal components of the tumor microenvironment and can precipitate several cancers.[27] In addition, M2 macrophages are known to augment the invasive ability of colon cancer cells through matrix metalloproteinases.[28] More importantly, M2 macrophage-derived exosomes were previously documented to promote the migration and invasion of colon cancer cells,[12] which is in accordance with our findings. Furthermore, we uncovered that miR-155-5p was highly expressed in M2 macrophage-derived exosomes, whereby M2 macrophage-derived exosomal miR-155-5p could promote the proliferation and antiapoptosis of colon cancer cells. Similarly, a prior study also found upregulated expression of miR-155-5p in M2 macrophage-derived exosomes and found that miR-155-5p was transferred to colorectal cancer cells via M2 macrophage-derived exosomes.[12] Meanwhile, macrophage-derived exosomal miR-21, another miRNA, is known to lead to the enhancement of gastric cancer cell apoptosis.[14] Prior studies have also shown that macrophage-derived exosomal miR-155 can promote fibroblast inflammation in cardiac injury,[13] whereas miR-155-5p can also positively regulate the migration of colon cancer cells through posttranscriptional regulation of Human Antigen R.[19] Besides, miR-155 has been previously elucidated to possess the ability to enhance the invasiveness of colorectal cancer SW-480 cells via Wnt/β-catenin regulation.[29] Further in line with our results, another study revealed that inhibition of miR-155 can bring about a reduction in cell proliferation and an elevation in cell apoptosis in colon cancer.[30] Our findings in conjunction with existing evidence indicate that that M2 macrophage-derived exosomal miR-155-5p could promote the proliferation and antiapoptosis ability of colon cancer cells.

Moreover, another critical finding in the current study was that the transfer of miR-155-5p into colon cancer cells via M2 macrophage-derived exosomes to target ZC3H12B diminished the expression levels of IL-6. ZC3H12B is regarded as the most active member of the ZC3H12 family, by virtue of its involvement in processes such as inflammation and degradation of inflammatory mRNAs.[21] In addition, the ZC3H12 family also negatively mediates the activation of macrophages, emphasizing its involvement in host immunity and inflammatory diseases.[31] Previous studies have also shown that downregulation of ZC3H12A can augment the aggressive features of tumor and serves as a contributing factor to the decline in disease-free survival of colorectal cancer patients.[32] On the other hand, IL-6 can induce STAT3 phosphorylation in colon cancer cells with overexpressed RAB3C, which promotes the migration of colon cancer cells.[33] More importantly, ZC3H12B binds to the IL-6 mRNA, decreasing the production of IL-6 protein upon stimulation with IL-1β, which is in accordance with our findings.[21] Even more so, a prior study found that miR-155-5p amplified the expression of IL-6 in thyroid follicle cells and also increased the IL-6 expression in hyperlipidemia and patients with familial hypercholesterolemia.[34,35] Additionally, our findings revealed that M2 macrophage-derived exosomal miR-155-5p promoted the IL-6 expression by downregulating the ZC3H12B expression, thus inhibiting T cell immune response and promoting immune escape. Consistent with our findings, IL-6 has also been previously shown to regulate immune cells, including T cells and macrophages.[36] Furthermore, IL-6 is known to stimulate tumor escape from immune surveillance induced by cell-in-cell structures that were formed by lymphocytes and colon cancer cells.[37]

Altogether, findings obtained in our study concluded that M2 macrophage-derived exosomes transferred miR-155-5p into colon cancer cells to target ZC3H12B, which increased IL-6 expression and then promoted immune escape and tumor formation in colon cancer (Figure 7). Our findings provide new insight that can be applied in the treatment of colon cancer. However, we recognize that additional mechanisms may be in place to regulate such pathways, which warrants further exploration. Moreover, future studies should be conducted on the effect of M2 macrophage-derived exosomes on normal colon cells.

MATERIALS AND METHODS

Ethics statement

The current study was approved by the Ethics Committee of Shanghai Tenth People's Hospital, Tongji University School of Medicine, and

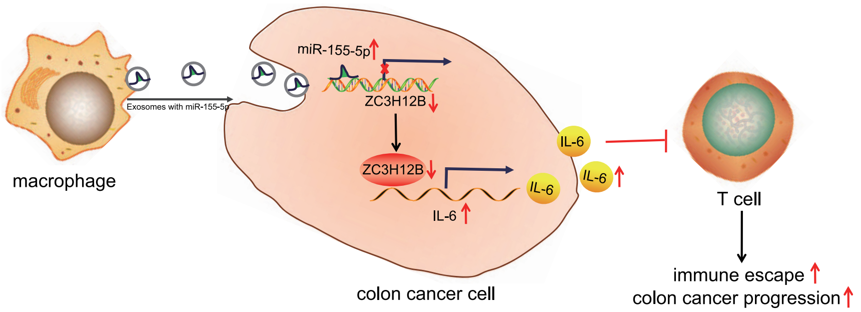

Figure 7. The mechanism of M2 macrophage-derived exosomal miR-155-5p and ZC3H12B in colon cancer
M2 macrophage-derived exosomal miR-155-5p went into colon cancer cells to downregulate ZC3H12B and to upregulate IL-6, which inhibited T cell immune response, thus promoting immune escape in colon cancer.

adhered to the Declaration of Helsinki. Signed informed consent was obtained from all participants prior to enrollment. All animal experiments were performed with the approval of the Animal Ethics Committee of Shanghai Tenth People's Hospital, Tongji University School of Medicine, and extensive efforts were made to ensure minimal suffering in the included animals.

Patient enrollment

Colon tissue samples were collected from 36 patients with colon cancer (23 males, 13 females; calculated mean age of 56.44 ± 14.28 yr) from January 2017 to January 2019 at the Shanghai Tenth People's Hospital, Tongji University School of Medicine. In addition, corresponding adjacent tissues were also obtained from the patients and used as normal controls. None of the included patients underwent radiation or chemotherapy prior to sample collection. All samples were confirmed according to the histological classification and classification criteria for colon cancer issued by the World Health Organization and again pathologically confirmed.

Cell treatment

Colon cancer cell line (SW48) cells and human normal colonic epithelial cells (CCD841CoN) were procured from American Type Culture Collection (Rockville, MD, USA). M1 and M2 macrophages were subsequently isolated from the colon cancer tissues by a previously described method.[38,39] In brief, tumor tissues were sliced and added with 10 μL of Liberase DL stock solution 28 U/mL, 20 μL of Liberase TL stock solution 14 U/mL, and 10 μL of DNase I (15 mg/mL stock solution 100×)/mL for digestion. After centrifugation, fluorescence-activated cell sorting (FACS) staining, and cell sorting, the CD163-positive and CD206-positive cells (tumor-related-M2-like macrophages) and CD14-positive and CD86-positive cells (tumor-related-M1-like macrophages) were collected. Briefly, colon cancer tissues were washed with PBS, sliced into small pieces (1 mm^3) with a surgical blade, and transferred to a 15-mL centrifuge tube, followed by the addition of 1 mL of PBS, with a final volume of 3 mL. Next, DL collagenase, TL collagenase, and DNase were added, and incubation was carried out at 37°C for 45 min, after which the digestion was terminated with the addition of staining buffer. The cell suspension was then filtered through a cell strainer and centrifuged for 5 min (500 × *g*, 4°C). The tube was added with 1 mL of staining buffer and 3 mL of erythrocyte lysate, followed by an ice bath for 10 min. Subsequently, the cell suspension was added with 30 mL of staining buffer and centrifuged for 5 min (500 × *g*, 4°C). The supernatant was then discarded and the tube was added with 900 μL of staining buffer, PBS supplemented with 10% human AB serum, followed by an ice bath for 30 min. After the ice bath, the tube was added with 5 mL of staining buffer, filtered, and centrifuged (500 × g, 4°C; 5 min). The supernatant was discarded, and the tube was added with 1 mL of staining buffer. Immunostaining and western blot analysis were performed to determine the expression patterns of CD163, CD68, and CD206.

The cells were cultured in Dulbecco's modified Eagle's medium (DMEM) (Gibco, Grand Island, NY, USA) containing 10% fetal bovine serum (FBS), 100 U/mL penicillin, and 100 μg/mL streptomycin in a humidified incubator at 37°C with 5% CO_2 in air. Cells were detached with the use of 0.25% trypsin and passaged at the rate of 1:3.

In addition, peripheral blood mononuclear cells (PBMCs) were isolated from healthy donor and colon cancer patients by density gradient centrifugation using Ficoll-Paque Plus. Blood neutrophils were harvested after red blood cells were lysed with a lysis solution. T cells from PBMCs were subsequently purified by using anti-CD3 magnetic beads to obtain $CD3^+$ T cells.

Exosome isolation, purification, and identification

The M2 macrophage culture medium was ultracentrifuged to collect exosomes. The conditioned medium was centrifuged at 500 × *g* for 15 min, at 3,000 × *g* for 15 min, and at 12,000 × *g* for 30 min at 4°C to remove the cells and debris. The medium was further centrifuged at 140,000 × *g* for 80 min to purify the exosomes. After resuspension in PBS, the previous ultracentrifugation was performed once again to further purify the exosomes.

Nanoparticle tracking analysis

A total of 20 μg of exosomes was dissolved in 1 mL of PBS and vortexed for 1 min to evenly distribute the exosomes. The distribution size of exosomes was directly observed with a NanoSight Nanoparticle Tracking Analyzer (Malvern Instruments, Malvern, UK).

Transmission electron microscope

The prepared exosomes were immediately fixed in 4% glutaraldehyde for 2 h (4°C) and in 1% osmium tetroxide for 2 h. Exosomes were then dehydrated with ethanol and acetone gradients, immersed in epoxy resin, paraffin embedded, and polymerized. Semithin sections

(0.5 μm) and ultrathin sections (60 nm) were subsequently prepared under an optical microscope. Finally, the exosomes were stained with uranium acetate and lead citrate and observed under an electron microscope.

DiO-labeled exosome transfer assay

A total of 1,000 μL of the exosomal suspension was added with 10 μL of DiO solution and incubated at 37°C for 30 min. SW48 cells were cocultured with 25 mg of M2 macrophage-derived exosomes labeled with DiO (green) for 24 h. In addition, SW48 cells were also incubated with DiO-unlabeled exosomes for 24 h as a NC. Next, the exosomes were fixed, followed by nuclei staining. Finally, the exosomes were observed under a laser confocal fluorescence microscope, and images were acquired.

Plasmid transfection

M2 macrophages were transfected with plasmids expressing inhibitor NC (NC of miR-155-5p inhibitor) or miR-155-5p inhibitor.

Meanwhile, SW48 cells were transfected with plasmids expressing mimic NC (NC of miR-155-5p mimic), miR-155-5p mimic, inhibitor NC, miR-155-5p inhibitor, sh-NC (scramble shRNA control), sh-ZC3H12B, oe-NC (empty virus vector), oe-ZC3H12B, oe-IL-6, oe-ZC3H12B + oe-IL-6, miR-155-5p mimic + oe-NC, or miR-155-5p mimic + oe-ZC3H12B.

The miR-155-5p mimic and miR-155-5p inhibitor and their controls were purchased from Guangzhou RiboBio (Guangdong, P.R. China). Upon reaching 85–90% cell confluence, M2 macrophages or SW48 colon cancer cells were transfected with plasmids (80 nM) according to the manuals for Lipofectamine 2000 reagents (Invitrogen, Carlsbad, CA, USA). pGCSIL-PUR lentivirus encoding human ZC3H12B (sh-ZC3H12B) and Lenti-OE lentivirus overexpressing IL-6 or ZC3H12B were purchased from Shanghai Genechem (Shanghai, P.R. China). After 6 h of transfection the medium was renewed, and the cells were collected for subsequent experiments after 48 h.

Cy3-labeled miR-155-5p was transfected into M2 macrophages with the Lipofectamine 2000 reagent (Invitrogen). Macrophages containing Cy3-miR-155-5p were cocultured with SW48 cells and observed and detected with a fluorescence microscope and flow cytometry.

qRT-PCR

Total cellular RNAs were extracted with the TRIzol reagent (Catalog No. 16096020, Thermo Fisher Scientific, Waltham, MA, USA). For miRNA, poly(A) tail PCR kits (b532451, Shanghai Sangon Biotechnology, Shanghai, China; including universal PCR primer R and U6 universal PCR primer R) were employed to obtain the cDNA of miRNA containing poly(A) tail. Non-miRNA reverse transcription was performed in accordance with the instructions of the cDNA reverse transcription kit (K1622, Beijing Yaanda Biotechnology, Beijing, P.R. China). PCR amplification of the target gene was conducted with a 25-μL system, including 300 ng of cDNA, 1× PCR buffer, 200 μmol/L deoxyribonucleotide triphosphates (dNTPs), 80 pmol/L forward primers, 80 pmol/L reverse primers, and 0.5 U of Taq enzyme (S10118, Shanghai YuanYe Bio-Technology, Shanghai, P.R. China). The reaction conditions were as follows: predenaturation at 94°C for 5 min, 30 cycles of denaturation at 94°C for 30 s, annealing at 54.5°C for 30 s and extension at 72°C for 30 s, and finally extension at 72°C for 10 min. The primers used in the reaction are shown in Table S1. U6 was utilized as the loading control of miR-155-5p, and glyceraldehyde-3-phosphate dehydrogenase (GAPDH) served as the internal reference for other genes. The fold changes were calculated by means of relative quantification (the $2^{-\Delta\Delta Ct}$ method).

Western blot analysis

Tissue or total cell protein content was extracted with phenylmethanesulfonyl fluoride (PMSF) and protease inhibitors. After separation by polyacrylamide gel electrophoresis, the proteins were electrotransferred onto a polyvinylidene fluoride (PVDF) membrane that was blocked with 5% bovine serum albumin (BSA). Next, the membrane was blocked at room temperature for 1 h. Primary antibodies against CD63 (dilution ratio of 1:1,000, ab216130), CD81 (dilution ratio of 1:1,000, ab109201), ZC3H12B (dilution ratio of 1:1,000, ab106710), IL-6 (dilution ratio of 1:1,000, ab6672), and GAPDH (dilution ratio of 1:2500, ab9485) were added to the membrane for incubation at 4°C overnight. The following day, horseradish peroxidase (HRP)-labeled goat anti-rabbit immunoglobulin G (IgG; dilution ratio of 1:20,000, ab205718) was added to the membrane for further incubation for 1.5 h at room temperature. All the aforementioned antibodies were purchased from Abcam (Cambridge, UK). The developing solution (NCI 4106, Pierce, Rockford, IL, USA) was then added to visualize the membrane. Protein quantification was performed with ImageJ 1.48u software (Bio-Rad, Hercules, CA, USA), and relative protein expression was expressed as the ratio of the gray value of target protein bands to the protein band of GAPDH.

Coculture of M2 macrophage and its exosomes with colon cancer cells

In order to inhibit exosomal release from macrophages, macrophage cells were treated with the exosome inhibitor GW4869. Subsequently, the macrophages were plated in 6-well plates (density of 1×10^6 cells/well), and upon reaching 80–90% confluence the cells were treated with 10% GW4869 (D1692-5MG, Sigma-Aldrich, St. Louis, MO, USA) or 0.005% DMSO as control. After 24 h of treatment, the cells and the supernatant were collected.

M2 macrophages were plated in the basolateral chamber of a 24-well Transwell chamber (density of 1×10^4 cells/well), and the apical chamber was seeded with SW48 colon cancer cells . The insert pore size between the apical and basolateral chambers was 0.4 μm. After 24 h of coculture, SW48 colon cancer cells were isolated and tested for cell proliferation and apoptosis.

Coculture of colon cancer cells with T cells *in vitro*

Carboxyfluorescein diacetate succinimidyl ester (CFSE)-labeled $CD3^+$ T cells were seeded into a 96-well plate (density of 1×10^5

cells). Subsequently, the T cells were cocultured with SW48 cells treated with different M2 macrophage-derived exosomes in medium containing rhIL-2 (20 IU/mL), anti-CD3 (2 μg/mL), and anti-CD28 (1 μg/mL) antibodies. Finally, the T cells were collected for flow cytometry after 48 h of culture.

Flow cytometry for apoptosis

After 48 h of transfection the cells were collected, and the cell concentration was adjusted to 1×10^6 cells/mL. Next, the cells were fixed with precooled 70% ethanol solution and allowed to stand overnight at 4°C. A total of 100 μL of cell suspension was centrifuged (cells not less than 1×10^6 cells/mL) and then resuspended in 200 μL of binding buffer. A total of 10 μL of Annexin V-fluorescein isothiocyanate (FITC) and 5 μL of propidium iodide (PI) were subsequently added, mixed with the cell suspension gently, and reacted under dark conditions at room temperature for 15 min. Afterward, 300 μL of binding buffer was added to the cells, and a flow cytometer was used to detect apoptosis at a wavelength of 488 nm: $FITC^-/PI^-$: living cells; $FITC^+/PI^-$: early apoptotic cells; $FITC^+/PI^+$: late apoptotic cells; $FITC^-/PI^+$: necrotic cells. Early apoptotic cells and late apoptotic cells were regarded as apoptotic cells.

Flow cytometry for detection of cell surface antigen

The cells were made into a single-cell suspension and resuspended in staining buffer (BD Biosciences, Franklin Lakes, NJ, USA). T cells were cultured with PerCP-CD3 (1:100, BioLegend, San Diego, CA, USA, #100326, Armenian hamster) and fixed and permeabilized with Pacific blue-IFN-γ (1:50, BioLegend, San Diego, CA, USA, #505817, rat). Subsequently, the cells were detected with a BD FACS Canto II flow cytometer (BD Immunocytometry Systems, San Jose, CA, USA) and analyzed with FlowJo software.

EdU assay

EdU solution was added to the cell culture plate (cell medium and EdU solution were mixed at a ratio of 1,000:1), and the cells were incubated for 2 h at room temperature. Next, the cells were fixed with 40 g/L paraformaldehyde for 30 min and incubated for 8 min in glycine solution. The cells were then rinsed with PBS containing 0.5% Triton X-100. After rinsing, Apollo staining reaction solution was added to the cell culture plate, and the cells were incubated for 30 min at room temperature under dark conditions. Later, Hoechst 3334 reaction solution was added, and the cells were incubated another time for 20 min at room temperature in dark conditions. The cells were subsequently observed under a fluorescence microscope, and red light was utilized to photograph the cells with a 550-nm excitation channel. The red-stained cells were regarded as the proliferating cells. In addition, violet light was used to photograph the cells with a 350-nm excitation channel, and the blue-stained cells were total cells. Three fields of view were selected under a 400-fold field of view, and EdU-stained cells (proliferating cells) and Hoechst 33342-stained cells (total cells) were counted. Cell proliferation rate (%) = number of proliferating cells/total number of cells × 100.

Nude mouse xenograft model

A total of 20 female nonobese diabetic/severe combined immunodeficient (NOD/SCID) mice (Shanghai SLAC Laboratory Animal, Shanghai, P.R. China, aged 4–8 weeks) were procured and randomly divided into four groups, with five mice in each group. SW48 cells were transfected with oe-ZC3H12B or oe-NC, and M2 macrophages were transfected with miR-155-5p inhibitor or inhibitor NC. The SW48 cells were then cocultured with exosomes from transfected M2 macrophages. Subsequently, SW48 cells (5×10^5) were injected into the tail vein of female NOD/SCID mice with a superfine syringe. Later, a total of 10 mg of M2 macrophage-derived exosomes was injected into the tail vein every 3 days. The polyclonal T cells (5×10^6) [identified with 2 mg/mL anti-CD3 (ab135372) and 1 mg/mL anti-CD28 (ab243228) (Abcam)] were cocultured with transfected SW48 cells for 24 h and injected into the peritoneum of mice 10 days after inoculation of the SW48 cells.

Tumor size was measured once every 2 days with a vernier caliper, while tumor volume was calculated from three vertical measurements. After the mice were anaesthetized with pentobarbital sodium (57-33-0, Shanghai Bituo Biotechnology, Shanghai, P.R. China) at 50 mg/kg and euthanized, the tumors were excised, and the spleens were separated into single cells for flow cytometry.

Dual luciferase reporter gene assay

The 3¿ UTR dual luciferase reporter gene vector of ZC3H12B and the mutant plasmid with mutation of the binding site between ZC3H12B and miR-155-5p, namely, PmirGLO-ZC3H12B-WT and PmirGLO-ZC3H12B-MUT, were constructed. The reporter plasmid was cotransfected into HEK293T cells with miR-155-5p mimic or mimic NC. After 24 h of transfection, the cells were lysed. Subsequently, the obtained cells were centrifuged at 12,000 rpm for 1 min, with the supernatant collected. Luciferase activity was tested with a dual luciferase reporter system (Dual-Luciferase Reporter Assay System, E1910, Promega, Madison, WI, USA).

The IL-6 mRNA 3¿ UTR reporter gene plasmid (pmirGLO_IL-6 3¿-UTR) was constructed to determine the binding relationship between ZC3H12B and IL-6.

mRNA degradation detection

First, SW48 cells were transfected with sh-ZC3H12B. After 24 h, RNA transcription was terminated with the addition of 10 μg/mL actinomycin D. Next, the total RNAs were extracted from the cells treated for 0, 15, 30, and 45 min, respectively, to measure the IL-6 mRNA content. At the time of analysis, the initial mRNA amount of each group of cells at 0 min was set to 100%, and quantitative calculation was conducted with a ratio of initial mRNA amount to content values at the corresponding 0 min.

ELISA

The collected cell supernatant was assayed for cytokine expression patterns according to the protocols of the IL-6 ELISA kit (human: ab46042, mouse: ab100713, Abcam). The optical density (OD) values

were measured at a wavelength of 450 nm with a versatile microplate reader (Synergy 2, BioTek, Winooski, VT, USA) by zeroing the blank control well. The standard product concentration was plotted on the x axis, and the OD value was plotted on the y axis. The regression equation of the standard curve was subsequently calculated, and the sample OD value was substituted into the equation to calculate the target protein concentration in the sample.

Statistical analysis

Statistical analyses were performed with SPSS 21.0 software (IBM, Armonk, NY, USA). Measurement data conforming to normal distribution and homogeneity of variance were expressed as mean ± standard deviation. Data within groups were analyzed by paired t test, while data were compared between two groups by independent-sample t test. Comparisons among multiple groups were analyzed by one-way analysis of variance (ANOVA), followed by Tukey's post hoc test. The volume of tumor at different time points was compared by Bonferroni-corrected repeated-measures ANOVA. The residual amount of IL-6 at different time points was analyzed by two-way ANOVA. A value of $p < 0.05$ was regarded as statistically significant.

SUPPLEMENTAL INFORMATION

Supplemental Information can be found online at https://doi.org/10.1016/j.omto.2021.02.005.

ACKNOWLEDGMENTS

The authors thank the reviewers for their helpful comments. This study was supported partly by grants from the National Natural Science Foundation of China (81772932, 81972214, 81472202, 81201535, and 81302065); Scientific Research Fund Project of Anhui Medical University (2018xkj058); Construction of Clinical Medical Center for Tumor Biological Samples in Nantong (HS2016004); The Peak of Six Personnel Foundation in Jiangsu Province (WSW-009); Jiangsu 333 Program (BRA2017205); Nantong Medical Key Talents Training Plan (Key 43); Nantong Science and Technology Project (yyz15026); and Wu Jieping Medical Foundation (320.6750.14326).

AUTHOR CONTRIBUTIONS

Conceptualization: Y.S.M., J.B.L., and D.F.; data curation: P.S.C. and T.M.W.; formal analysis: L.P.G. and J.Z.; investigation: Y.S.M., L.L., and H.M.W.; methodology: Z.J.W., C.C.L., and W.L.; project administration: D.F.; resources: G.R.W. and H.X.; software: F.Y.; supervision: J.B.L. and Q.L.L.; validation: Q.L.L. and T.M.W.; visualization: F.Y. and C.C.L.; writing – original draft: Y.S.M., P.S.C., L.P.G., L.L., Z.J.W., G.R.W., J.B.L., and D.F.; writing – revising: T.M.W., C.C.L., F.Y., J.Z., H.M.W., H.X., W.L., and Q.L.L.; writing – review: Y.S.M., T.M.W., C.C.L., F.Y., J.Z., P.S.C., L.P.G., H.M.W., H.X., L.L., Z.J.W., G.R.W., W.L., Q.L.L., J.B.L., and D.F.

DECLARATION OF INTERESTS

The authors declare no competing interests.

REFERENCES

1. Chien, C.W., Hou, P.C., Wu, H.C., Chang, Y.L., Lin, S.C., Lin, S.C., Lin, B.W., Lee, J.C., Chang, Y.J., Sun, H.S., and Tsai, S.J. (2016). Targeting TYRO3 inhibits epithelial-mesenchymal transition and increases drug sensitivity in colon cancer. Oncogene *35*, 5872–5881.
2. Vermeer, N.C.A., Claassen, Y.H.M., Derks, M.G.M., Iversen, L.H., van Eycken, E., Guren, M.G., Mroczkowski, P., Martling, A., Johansson, R., Vandendael, T., et al. (2018). Treatment and Survival of Patients with Colon Cancer Aged 80 Years and Older: A EURECCA International Comparison. Oncologist *23*, 982–990.
3. Gangireddy, V.G.R., Coleman, T., Kanneganti, P., Talla, S., Annapureddy, A.R., Amin, R., and Parikh, S. (2018). Polypectomy versus surgery in early colon cancer: size and location of colon cancer affect long-term survival. Int. J. Colorectal Dis. *33*, 1349–1357.
4. Zhao, H., Zhang, N., Ho, V., Ding, M., He, W., Niu, J., Yang, M., Du, X.L., Zorzi, D., Chavez-MacGregor, M., and Giordano, S.H. (2018). Adherence to treatment guidelines and survival for older patients with stage II or III colon cancer in Texas from 2001 through 2011. Cancer *124*, 679–687.
5. Zeng, Z., Yang, H., and Xiao, S. (2018). ACTL6A expression promotes invasion, metastasis and epithelial mesenchymal transition of colon cancer. BMC Cancer *18*, 1020.
6. Ma, Y.S., Huang, T., Zhong, X.M., Zhang, H.W., Cong, X.L., Xu, H., Lu, G.X., Yu, F., Xue, S.B., Lv, Z.W., and Fu, D. (2018). Proteogenomic characterization and comprehensive integrative genomic analysis of human colorectal cancer liver metastasis. Mol. Cancer *17*, 139.
7. Duan, S., Guo, W., Xu, Z., He, Y., Liang, C., Mo, Y., Wang, Y., Xiong, F., Guo, C., Li, Y., et al. (2019). Natural killer group 2D receptor and its ligands in cancer immune escape. Mol. Cancer *18*, 29.
8. Fletcher, R., Wang, Y.J., Schoen, R.E., Finn, O.J., Yu, J., and Zhang, L. (2018). Colorectal cancer prevention: Immune modulation taking the stage. Biochim. Biophys. Acta Rev. Cancer *1869*, 138–148.
9. Müller, J., von Bernstorff, W., Heidecke, C.D., and Schulze, T. (2017). Differential S1P Receptor Profiles on M1- and M2-Polarized Macrophages Affect Macrophage Cytokine Production and Migration. BioMed Res. Int. *2017*, 7584621.
10. Zhang, L.L., Zhang, L.F., and Shi, Y.B. (2018). Down-regulated paxillin suppresses cell proliferation and invasion by inhibiting M2 macrophage polarization in colon cancer. Biol. Chem. *399*, 1285–1295.
11. Zhang, K., Xu, S., Shi, X., Xu, G., Shen, C., Liu, X., and Zheng, H. (2019). Exosomes-mediated transmission of foot-and-mouth disease virus in vivo and in vitro. Vet. Microbiol. *233*, 164–173.
12. Lan, J., Sun, L., Xu, F., Liu, L., Hu, F., Song, D., Hou, Z., Wu, W., Luo, X., Wang, J., et al. (2019). M2 Macrophage-Derived Exosomes Promote Cell Migration and Invasion in Colon Cancer. Cancer Res. *79*, 146–158.
13. Wang, C., Zhang, C., Liu, L., A, X., Chen, B., Li, Y., and Du, J. (2017). Macrophage-Derived mir-155-Containing Exosomes Suppress Fibroblast Proliferation and Promote Fibroblast Inflammation during Cardiac Injury. Mol. Ther. *25*, 192–204.
14. Zheng, P., Chen, L., Yuan, X., Luo, Q., Liu, Y., Xie, G., Ma, Y., and Shen, L. (2017). Exosomal transfer of tumor-associated macrophage-derived miR-21 confers cisplatin resistance in gastric cancer cells. J. Exp. Clin. Cancer Res. *36*, 53.
15. Li, B., Wang, S., and Wang, S. (2018). MiR-195 suppresses colon cancer proliferation and metastasis by targeting WNT3A. Mol. Genet. Genomics *293*, 1245–1253.
16. Oshima, G., Guo, N., He, C., Stack, M.E., Poon, C., Uppal, A., Wightman, S.C., Parekh, A., Skowron, K.B., Posner, M.C., et al. (2017). In Vivo Delivery and Therapeutic Effects of a MicroRNA on Colorectal Liver Metastases. Mol. Ther. *25*, 1588–1595.
17. Toiyama, Y., Okugawa, Y., Fleshman, J., Richard Boland, C., and Goel, A. (2018). MicroRNAs as potential liquid biopsy biomarkers in colorectal cancer: A systematic review. Biochim. Biophys. Acta Rev. Cancer *1870*, 274–282.
18. Qu, Y.L., Wang, H.F., Sun, Z.Q., Tang, Y., Han, X.N., Yu, X.B., and Liu, K. (2015). Up-regulated miR-155-5p promotes cell proliferation, invasion and metastasis in colorectal carcinoma. Int. J. Clin. Exp. Pathol. *8*, 6988–6994.

19. Al-Haidari, A., Algaber, A., Madhi, R., Syk, I., and Thorlacius, H. (2018). MiR-155-5p controls colon cancer cell migration via post-transcriptional regulation of Human Antigen R (HuR). Cancer Lett. *421*, 145–151.

20. Pathak, S., Meng, W.J., Nandy, S.K., Ping, J., Bisgin, A., Helmfors, L., Waldmann, P., and Sun, X.F. (2015). Radiation and SN38 treatments modulate the expression of microRNAs, cytokines and chemokines in colon cancer cells in a p53-directed manner. Oncotarget *6*, 44758–44780.

21. Wawro, M., Wawro, K., Kochan, J., Solecka, A., Sowinska, W., Lichawska-Cieslar, A., Jura, J., and Kasza, A. (2019). ZC3H12B/MCPIP2, a new active member of the ZC3H12 family. RNA *25*, 840–856.

22. Jolma, A., Zhang, J., Mondragón, E., Morgunova, E., Kivioja, T., Laverty, K.U., Yin, Y., Zhu, F., Bourenkov, G., Morris, Q., et al. (2020). Binding specificities of human RNA-binding proteins toward structured and linear RNA sequences. Genome Res. *30*, 962–973.

23. Chen, T.M., Huang, Y.T., and Wang, G.C. (2017). Outcome of colon cancer initially presenting as colon perforation and obstruction. World J. Surg. Oncol. *15*, 164.

24. Zeng, H., Yuan, F., Mi, Y., Xian, G., Qin, C., and Zhang, D. (2018). As an independent prognostic factor, USP6 promotes the invasion and metastasis of colon cancer. Biochem. Biophys. Res. Commun. *505*, 816–822.

25. Fadaka, A.O., Pretorius, A., and Klein, A. (2019). Biomarkers for Stratification in Colorectal Cancer: MicroRNAs. Cancer Contr. *26*, 1073274819862784.

26. Chouhan, H., Sammour, T., Thomas, M.L., and Moore, J.W. (2018). The interaction between BRAF mutation and microsatellite instability (MSI) status in determining survival outcomes after adjuvant 5FU based chemotherapy in stage III colon cancer. J. Surg. Oncol. *118*, 1311–1317.

27. Ngambenjawong, C., Gustafson, H.H., Pineda, J.M., Kacherovsky, N.A., Cieslewicz, M., and Pun, S.H. (2016). Serum Stability and Affinity Optimization of an M2 Macrophage-Targeting Peptide (M2pep). Theranostics *6*, 1403–1414.

28. Vinnakota, K., Zhang, Y., Selvanesan, B.C., Topi, G., Salim, T., Sand-Dejmek, J., Jönsson, G., and Sjölander, A. (2017). M2-like macrophages induce colon cancer cell invasion via matrix metalloproteinases. J. Cell. Physiol. *232*, 3468–3480.

29. Liu, N., Jiang, F., Han, X.Y., Li, M., Chen, W.J., Liu, Q.C., Liao, C.X., and Lv, Y.F. (2018). MiRNA-155 promotes the invasion of colorectal cancer SW-480 cells through regulating the Wnt/β-catenin. Eur. Rev. Med. Pharmacol. Sci. *22*, 101–109.

30. Pu, J., Bai, D., Yang, X., Lu, X., Xu, L., and Lu, J. (2012). Adrenaline promotes cell proliferation and increases chemoresistance in colon cancer HT29 cells through induction of miR-155. Biochem. Biophys. Res. Commun. *428*, 210–215.

31. Liang, J., Wang, J., Azfer, A., Song, W., Tromp, G., Kolattukudy, P.E., and Fu, M. (2008). A novel CCCH-zinc finger protein family regulates proinflammatory activation of macrophages. J. Biol. Chem. *283*, 6337–6346.

32. Chen, T., Du, D., Chen, J., Zhou, P., Weinstein, J.N., Yao, L., and Liu, Y. (2019). ZC3H12A Expression in Different Stages of Colorectal Cancer. Oncoscience *6*, 301–311.

33. Chang, Y.C., Su, C.Y., Chen, M.H., Chen, W.S., Chen, C.L., and Hsiao, M. (2017). Secretory RAB GTPase 3C modulates IL6-STAT3 pathway to promote colon cancer metastasis and is associated with poor prognosis. Mol. Cancer *16*, 135.

34. Li, H., Zhang, X., Gao, L., Min, J., Zhang, Y., Zhang, R., and Yang, Y. (2018). TNF-alpha is upregulated in subacute thyroiditis and stimulates expression of miR-155-5p in thyroid follicle cells. Discov. Med. *26*, 67–77.

35. Zhu, M., Wei, Y., Geißler, C., Abschlag, K., Corbalán Campos, J., Hristov, M., Möllmann, J., Lehrke, M., Karshovska, E., and Schober, A. (2017). Hyperlipidemia-Induced MicroRNA-155-5p Improves β-Cell Function by Targeting *Mafb*. Diabetes *66*, 3072–3084.

36. Tsukamoto, H., Fujieda, K., Senju, S., Ikeda, T., Oshiumi, H., and Nishimura, Y. (2018). Immune-suppressive effects of interleukin-6 on T-cell-mediated anti-tumor immunity. Cancer Sci. *109*, 523–530.

37. Wang, S., Li, L., Zhou, Y., He, Y., Wei, Y., and Tao, A. (2019). Heterotypic cell-in-cell structures in colon cancer can be regulated by IL-6 and lead to tumor immune escape. Exp. Cell Res. *382*, 111447.

38. Quero, L., Hanser, E., Manigold, T., Tiaden, A.N., and Kyburz, D. (2017). TLR2 stimulation impairs anti-inflammatory activity of M2-like macrophages, generating a chimeric M1/M2 phenotype. Arthritis Res. Ther. *19*, 245.

39. Fukui, S., Iwamoto, N., Takatani, A., Igawa, T., Shimizu, T., Umeda, M., Nishino, A., Horai, Y., Hirai, Y., Koga, T., et al. (2018). M1 and M2 Monocytes in Rheumatoid Arthritis: A Contribution of Imbalance of M1/M2 Monocytes to Osteoclastogenesis. Front. Immunol. *8*, 1958.

OMTO, Volume *20*

Supplemental information

M2 macrophage-derived exosomal microRNA-155-5p promotes the immune escape of colon cancer by downregulating ZC3H12B

Yu-Shui Ma, Ting-Miao Wu, Chang-Chun Ling, Fei Yu, Jie Zhang, Ping-Sheng Cao, Li-Peng Gu, Hui-Ming Wang, Hong Xu, Liu Li, Zhi-Jun Wu, Gao-Ren Wang, Wen Li, Qin-Lu Lin, Ji-Bin Liu, and Da Fu

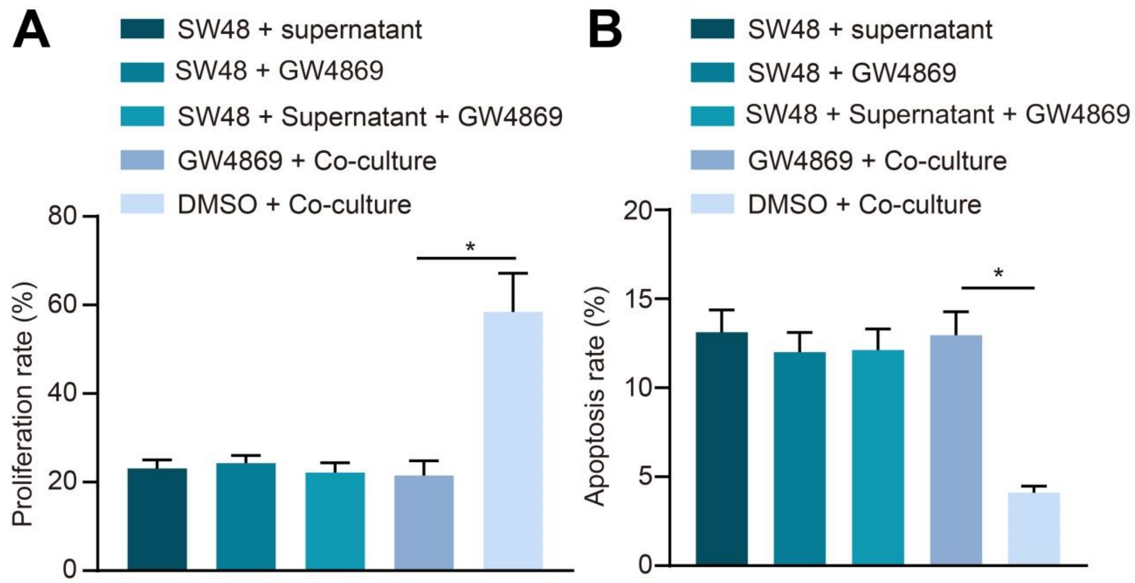

Figure S1. Inhibition of M2 macrophage-derived exosomes attenuated the proliferation and anti-apoptosis of SW48 cells. (A) EdU assay of SW48 cells proliferation after co-culture with M2 macrophages treated with GW4869. (B) Flow cytometric analysis of SW48 cell apoptosis after co-culture with M2 macrophages treated with GW4869.

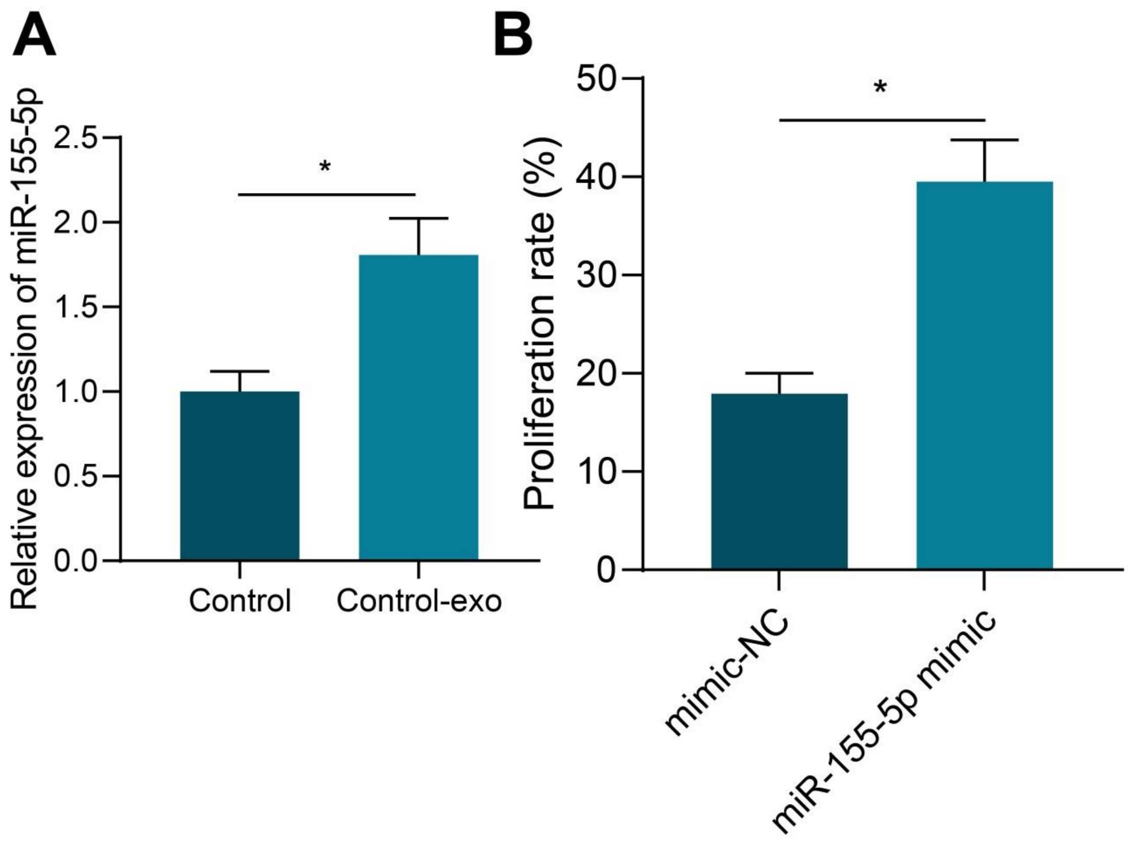

Figure S2. miR-155-5p augmented the proliferation of SW48 cells. (A) miR-155-5p expression determined by RT-qPCR in the receptor cells treated with exosomes isolated from untransfected M2 macrophages. (B) The proliferation of SW48 cells in response to miR-155-5p mimic determined by EdU staining.

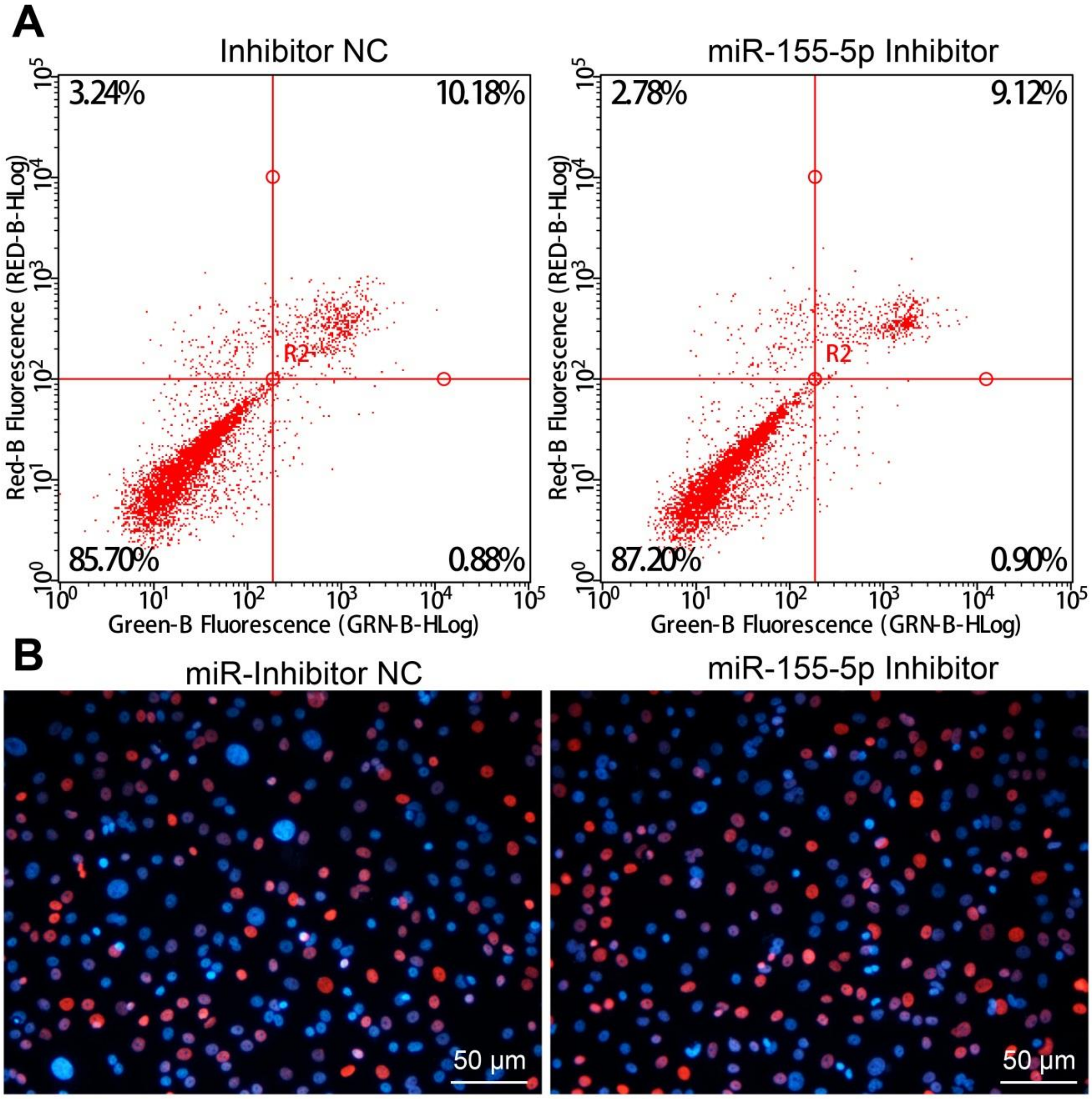

Figure S3. The proliferation and apoptosis of SW48 cells after inhibition of miR-155-5p. (A) The apoptosis of SW48 cells in response to exo-inhibitor NC or exo-miR-155-5p inhibitor determined by flow cytometry. (B) The proliferation of SW48 cells in response to exo-inhibitor NC or exo-miR-155-5p inhibitor determined by EdU staining.

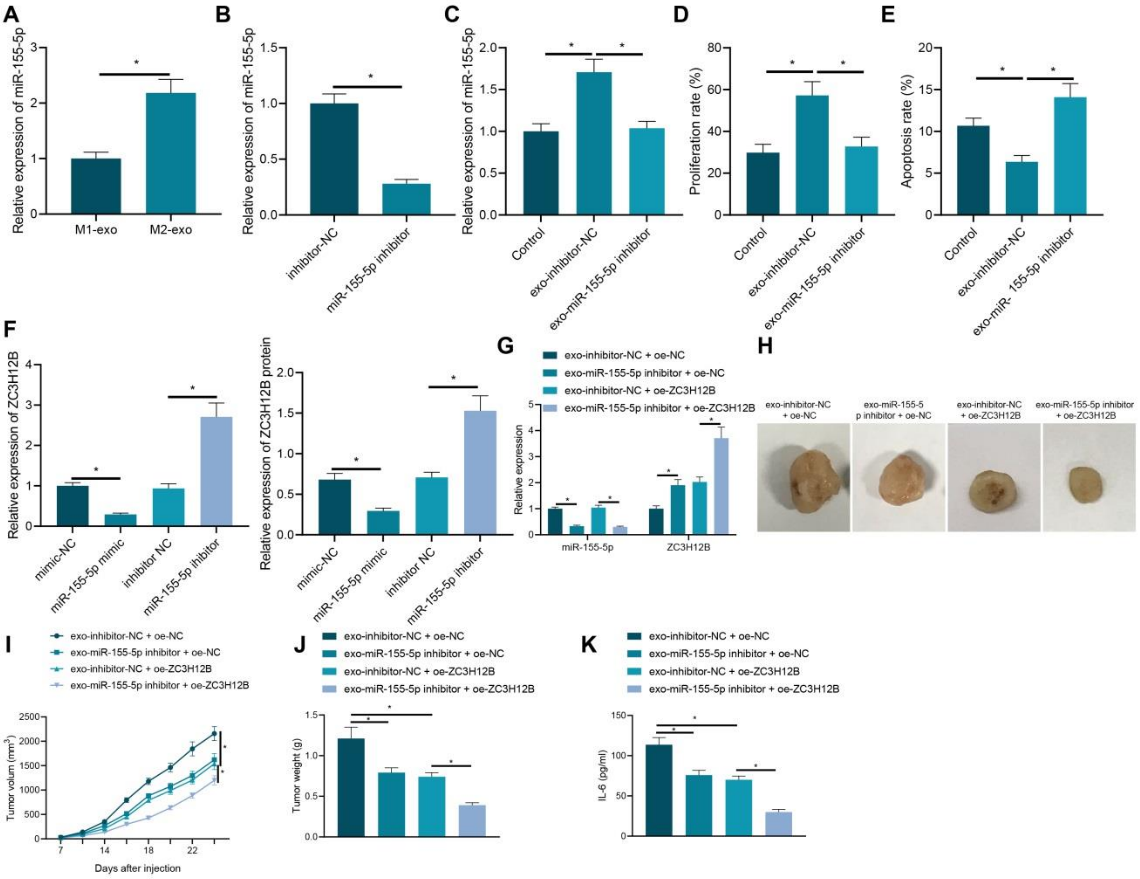

Figure S4. M2 macrophage-derived exosomal miR-155-5p promoted proliferation and repressed apoptosis of HT29 cells, as well as inhibiting immune escape *in vivo*. (A) miR-155-5p expression patterns determined by RT-qPCR in the exosomes from M1 and M2 macrophages. (B) miR-155-5p expression patterns determined by RT-qPCR in the exosomes from M2 macrophages transfected with miR-155-5p inhibitor. (C) miR-155-5p expression patterns determined by RT-qPCR in the HT29 cells co-cultured with the exosomes from M2 macrophages and transfected with miR-155-5p inhibitor. (D) HT29 cell proliferation measured by EdU assay upon treatment with exo-miR-155-5p inhibitor. (E) HT29 cell apoptosis measured by flow cytometry upon treatment with exo-miR-155-5p inhibitor. (F) ZC3H12B mRNA and protein expression patterns determined by RT-qPCR and Western blot analysis in SW48 cells following varied treatments. (G) RT-qPCR examining the expression patterns of miR-155-5p and ZC3H12B mRNA in tumor tissues from mice following varied treatments. (H) Representative images of tumors from mice following varied treatments. (I)

Tumor volume of mice. (J) Tumor weight of mice. (K) ELISA examining the expression patterns of IL-6 in spleen cell lysates of mice. The data were measurement data, and expressed as mean ± standard deviation. Comparisons between two groups were analyzed by independent sample *t* test. Comparisons among multiple groups were analyzed by one-way ANOVA, followed by Tukey's post hoc test. * $p < 0.05$. n = 5.

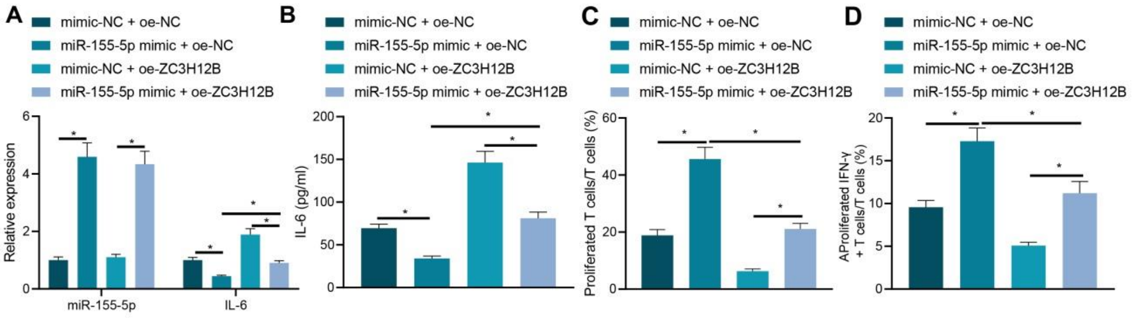

Figure S5. miR-155-5p functioned through IL-6 to regulate the ZC3H12B expression. (A) Expression patterns of miR-155-5p and IL-6 mRNA determined by RT-qPCR in the SW48 cells transfected with miR-155-5p mimic, oe-ZC3H12B or both. (B) IL-6 expression patterns determined by ELISA in the supernatants of SW48 cells transfected with miR-155-5p mimic, oe-ZC3H12B or both. (C) Proliferation of $CD3^+$ T cells determined by flow cytometry upon co-culture with SW48 cells transfected with miR-155-5p mimic, oe-ZC3H12B or both. (D) Proportion of IFN-γ^+ T cells determined by flow cytometry upon co-culture with SW48 cells transfected with miR-155-5p mimic, oe-ZC3H12B or both.

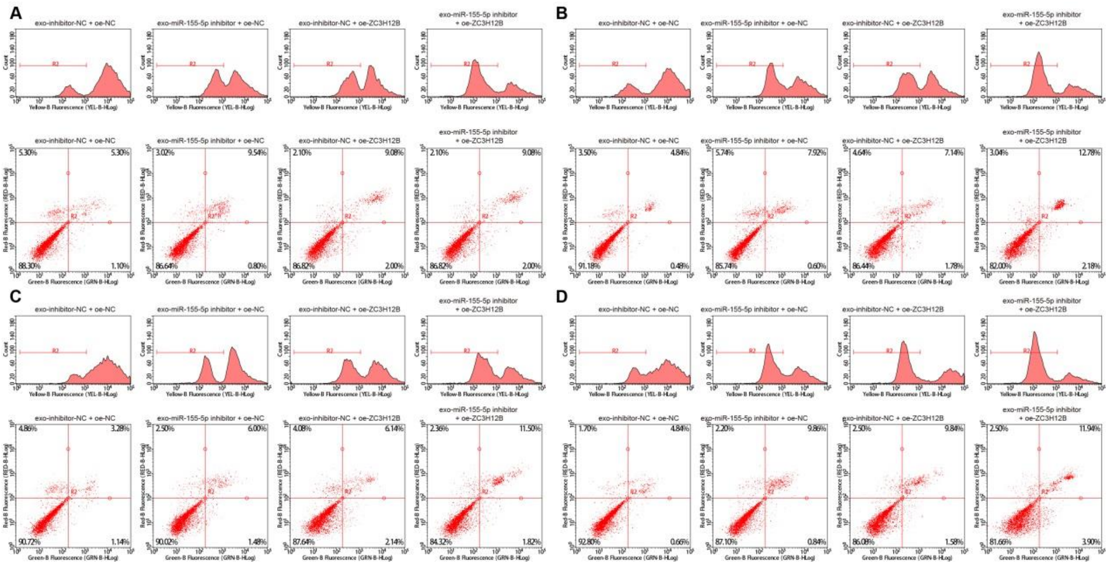

Figure S6. Measurement of expression patterns of T cell markers CD4 and CD8, M1 type markers CD14 and CD86, and M2 type markers CD163 and CD206 in the mouse tumor tissue. (A) CD4 expression patterns in mouse spleen tissues. (B) CD8 expression patterns in mouse spleen tissues examined by RT-qPCR. (C) CD14 and CD86 expression patterns in mouse spleen tissues examined by RT-qPCR. (D) CD163 and CD206 expression patterns in mouse spleen tissues examined by RT-qPCR.

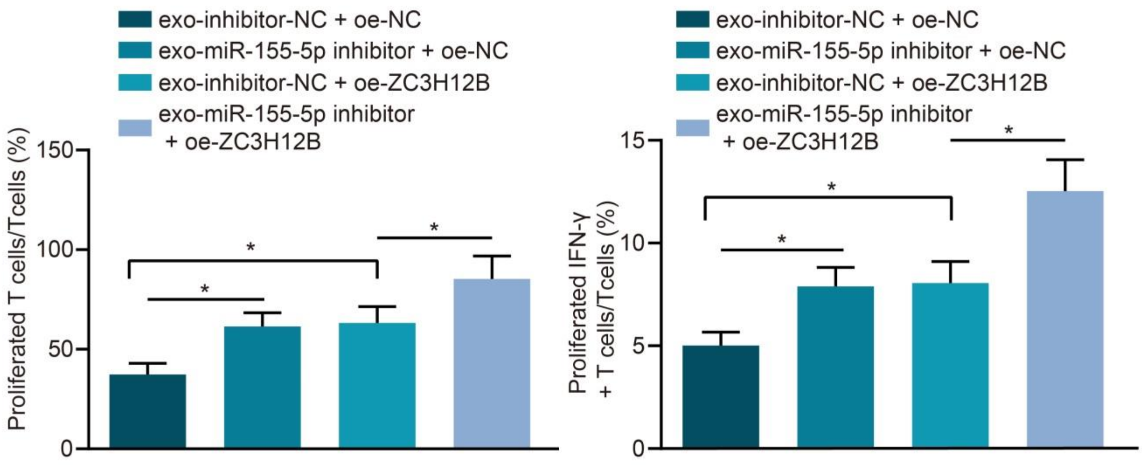

Figure S7. Flow cytometric analysis of the proportion of proliferated T cells and INF-γ^+ T cells in spleen cells of mice treated with exo-miR-155-5p inhibitor, oe-ZC3H12B or both.

Table S1 Primer sequences for RT-qPCR

Target	Primer sequence
miR-155-5p	F: 5’-TTAATGCTAATCGTGATAGGG-3’<br>R: 5’-GTGCAGGTCCGAGGT-3’
ZC3H12B	F: 5’-CCTCGTGGGCCCAGCTCCAG-3’<br>R: 5’-TCCCATGGCTCATTGCCACATTACT-3’
IL-6	F: 5'-GACAGCCACTCACCTCTTCA-3’<br>R: 5'-AGTGCCTCTTTGCTGCTTTC-3’
U6	F: 5'-CTCGCTTCGGCAGCACA-3’<br>R: 5'-AACGCTTCACGAATTTGCGT-3’
GAPDH	F: 5’-GTCAACGGATTTGGTCTGTATT-3’<br>R: 5’-CGCUUCACGAAUUUGCGUGUCAU-3’

Note: RT-qPCR, reverse-transcription quantitative polymerase chain reaction; miR-155-5p, microRNA-155-5p; IL-6, interleukin 6; GAPDH, glyceraldehyde-3-phosphate dehydrogenase; F, forward; R, reverse.
